# Preventing pancreatic fistula following pancreatectomy: meta-analysis of randomized clinical trials

**DOI:** 10.1093/bjsopen/zrag088

**Published:** 2026-09-21

**Authors:** Roberto M Montorsi, Alessio Marchetti, Tessa E Hendriks, Freek Daams, Olivier R Busch, Bas Groot Koerkamp, Matteo De Pastena, Hjalmar C van Santvoort, Bert A Bonsing, Salvatore Paiella, Giuseppe Malleo, Marc G Besselink, Roberto Salvia

**Affiliations:** Department of General and Pancreatic Surgery, The Pancreas Institute, University of Verona Hospital Trust, Verona, Italy; Department of Surgery, Amsterdam UMC, Location University of Amsterdam, Amsterdam, the Netherlands; Cancer Center Amsterdam, Amsterdam, the Netherlands; Department of General and Pancreatic Surgery, The Pancreas Institute, University of Verona Hospital Trust, Verona, Italy; Department of Surgery, NewYork University Grossman School of Medicine, New York, New York, USA; Department of Surgery, Amsterdam UMC, Location University of Amsterdam, Amsterdam, the Netherlands; Cancer Center Amsterdam, Amsterdam, the Netherlands; Department of Surgery, Leiden University Medical Center, Leiden, the Netherlands; Dutch Institute for Clinical Auditing, Leiden, the Netherlands; Cancer Center Amsterdam, Amsterdam, the Netherlands; Department of Surgery, Amsterdam UMC, Location Vrije Universiteit, Amsterdam, the Netherlands; Department of Surgery, Amsterdam UMC, Location University of Amsterdam, Amsterdam, the Netherlands; Cancer Center Amsterdam, Amsterdam, the Netherlands; Department of Surgery, Erasmus MC Cancer Institute, Rotterdam, the Netherlands; Department of General and Pancreatic Surgery, The Pancreas Institute, University of Verona Hospital Trust, Verona, Italy; Department of Surgery, Regional Academic Cancer Center Utrecht, University Medical Center Utrecht/St Antonius Hospital Nieuwegein, Utrecht, the Netherlands; Department of Surgery, Leiden University Medical Center, Leiden, the Netherlands; Pancreatic Surgery Unit, Department of Engineering for Innovation Medicine (DIMI), University of Verona, Verona, Italy; Pancreatic Surgery Unit, Department of Surgery, Dentistry, Paediatrics and Gynecology, University of Verona, Verona, Italy; Pancreatic Surgery Unit, Department of Engineering for Innovation Medicine (DIMI), University of Verona, Verona, Italy; Pancreatic Surgery Unit, Department of Surgery, Dentistry, Paediatrics and Gynecology, University of Verona, Verona, Italy; Department of Surgery, Amsterdam UMC, Location University of Amsterdam, Amsterdam, the Netherlands; Cancer Center Amsterdam, Amsterdam, the Netherlands; Department of General and Pancreatic Surgery, The Pancreas Institute, University of Verona Hospital Trust, Verona, Italy; Pancreatic Surgery Unit, Department of Engineering for Innovation Medicine (DIMI), University of Verona, Verona, Italy

## Abstract

**Background:**

The majority of postoperative morbidity and mortality after partial pancreatectomy is attributable to postoperative pancreatic fistula. Several interventions to prevent postoperative pancreatic fistula have been assessed in randomized clinical trials but consensus on their efficacy is lacking. This is the first systematic review and meta-analysis of randomized trials focusing on strategies to prevent postoperative pancreatic fistula after all types of partial pancreatectomy.

**Methods:**

Eligible randomized clinical trials assessing postoperative pancreatic fistula following partial pancreatectomy were included from the Evidence Map of Pancreatic Surgery database (2005–2025). As primary endpoint, both the 2005 and 2016 International Study Group for Pancreatic Surgery definitions of grade B/C postoperative pancreatic fistula were accepted. Interventions shown to reduce postoperative pancreatic fistula in at least one randomized trial were identified, and meta-analysis undertaken for those with at least three available trials, irrespective of their outcome.

**Results:**

Overall, 193 RCTs were included with 29 408 patients from 24 countries. The pooled rate of postoperative pancreatic fistula grade B/C was 15.1%: 14.2% after pancreatoduodenectomy and 19.1% after left pancreatectomy. Overall, 24 trials identified 18 interventions as effective in reducing the rate of postoperative pancreatic fistula: 11 intraoperative and 7 postoperative. Eight interventions were eligible for meta-analysis, involving 46 trials. Following meta-analysis, three interventions remained effective: drain omission in left pancreatectomy, perioperative use of somatostatin analogues, and administration of perioperative corticosteroids. The certainty of evidence was high for drain omission, moderate for somatostatin analogues, and low for corticosteroids. Eleven trials focused specifically on high-risk patients and four on ongoing postoperative pancreatic fistula. No trial combined interventions.

**Conclusion:**

Omission of drains in left pancreatectomy, perioperative use of somatostatin analogues, and administration of perioperative corticosteroids are effective strategies to prevent postoperative pancreatic fistula after partial pancreatectomy. However, current evidence remains immature and further RCTs evaluating these interventions are warranted, as they are likely to change the conclusions of subsequent meta-analyses. Such trials could potentially focus on high-risk patient cohorts and/or assess combinations of interventions.

## Teaser

This systematic review and meta-analysis explored the prevention and treatment of postoperative pancreatic fistula (POPF), and identified 18 interventions that reduced its incidence. After meta-analysis, three interventions remained effective: no drain placement in left pancreatectomy, perioperative somatostatin analogues, and perioperative cortisone. However, the certainty of evidence on these and numerous other interventions for prevention of POPF remains immature. Future RCTs are still likely to change the outcome of meta-analyses and could focus on high-risk patients and/or combine interventions.

## Introduction

Partial pancreatectomy remains one of the most challenging abdominal surgical procedures, as reflected by its high morbidity and mortality rates. Owing to improvements in patient selection, surgical technique, and postoperative management, the postoperative mortality rate in high-volume centres has decreased to 2.0–3.0%, whereas morbidity rates remain as high as 50.0%^1^. Nowadays, postoperative pancreatic fistula (POPF) remains the main driver of mortality and morbidity after partial pancreatectomy^2^.

Since the first internationally accepted definition of POPF and its subsequent update by the International Study Group for Pancreatic Surgery (ISGPS)^3,4^, multiple strategies have been investigated to prevent POPF following partial pancreatectomy. However, despite all these efforts, no consensus has been reached regarding the efficacy of these interventions. Furthermore, although the number of randomized clinical trials (RCTs) focusing on POPF is still growing, a comprehensive systematic review and meta-analysis including RCTs only is lacking.

The present study aimed to summarize current evidence on strategies to prevent POPF following partial pancreatectomy by means of a systematic review and meta-analysis of RCTs published since the definition of POPF was published in 2005.

## Methods

The systematic review protocol was registered in the International Prospective Register of Systematic Reviews (PROSPERO) on 1 December 2023 and was last updated on 6 August 2025 (registration number CRD42023490446)^5^. The systematic review protocol was written according to the PRISMA-P guidelines^6^. The present systematic review and meta-analysis was structured in accordance with the PRISMA guidelines^7^.

### Eligibility criteria

All RCTs related to pancreatic surgery published between 1 January 2005 and 31 July 2025 were reviewed from the ISGPS Evidence Map of Pancreatic Surgery database^8^. All RCTs reporting on POPF grade B/C, defined according to either the ISGPS 2005 or 2016 classification, were included. Prospective and retrospective studies, trial protocols, systematic reviews, meta-analyses, conference abstracts, secondary publications of previously published studies, letters, and commentaries were excluded. Publications without a full text available or written in languages other than English were excluded. Throughout the manuscript, only clinically relevant fistulas (grade B/C) were classified as POPF and analysed, whereas grade A/biochemical leaks were not considered.

### Information sources and search strategy

A systematic literature search was conducted using the ISGPS Evidence Map of Pancreatic Surgery database (a periodically updated platform; last searched on 31 July 2025). The search included all RCTs indexed under the sections ‘Partial pancreatoduodenectomy’, ‘Distal pancreatectomy’, ‘Various perioperative interventions’, and ‘Other surgical aspects’. The sections ‘Definitions and guidelines’, ‘Pancreatitis’, ‘Palliative measures’, and ‘Trauma’ were not explored, as they were not relevant to the objectives of the present review.

### Selection process

Two authors (A.M. and R.M.M.) independently undertook full-text screening using the aforementioned eligibility criteria. Disagreements were resolved by consensus and, if necessary, a third reviewer was consulted (M.G.B.).

### Data collection process

Two reviewers (A.M. and R.M.M.) extracted data independently from included studies using a standardized and pilot-tested data extraction form. Extracted data were cross-checked for accuracy. Discrepancies were resolved by consensus, with arbitration by a third reviewer (M.G.B.) when required. When necessary, corresponding authors were contacted to clarify missing or unclear data. No automated tools were used in the data extraction process.

The extracted data included: publication details (study title, publication date, authors, study design, ISGPS classification, risk of bias), baseline characteristics (number of patients, POPF risk, POPF risk/protective factors), clinical characteristics, intervention characteristics (non-invasive, endoscopic, angiographic, and surgical treatment) and postoperative characteristics (general and pancreas-specific complications).

### Outcomes

The primary outcome was the rate of POPF (grade B/C). In particular, an intervention was considered effective when associated with a significant reduction in POPF occurrence after partial pancreatectomy. Given the generally improving clinical care of patients over time, potentially leading to declining POPF rates, additional analyses evaluating temporal trends in POPF rates were undertaken with 5-year intervals.

Secondary outcomes included the identification and classification of RCTs focusing on POPF as a primary outcome according to target population (high-risk patients), study setting (ongoing POPF), and intervention strategy (combined intervention). Patients were classified as high-risk individuals if at least one of the following characteristics was present: high fistula risk score (FRS)^9^ or alternative FRS^10^, soft pancreas, parenchymal thickness at transection line (> 12 mm or > 14 mm), soft pancreas and main pancreatic duct (MPD) < 3 mm, non-dilated MPD alone, and histological presence of > 40.0% acini at frozen-section analysis. Additionally, factors evaluated within RCTs as potential risk factors for POPF were considered. For the RCTs included in meta-analyses, data on additional postoperative outcomes were also extracted, including delayed gastric emptying (DGE; ISGPS grade B/C)^11^, postpancreatectomy haemorrhage (PPH; ISGPS grade B/C)^12^, length of hospital stay (LOS), and mortality.

### Study risk-of-bias assessment

The quality of the included studies was assessed by two independent reviewers (A.M. and R.M.M.) using the Cochrane Risk of Bias 2 tool for RCTs (RoB-2)^13^. The assessment was undertaken only for studies included in the meta-analyses and for trials on interventions reporting a statistically significant reduction in POPF.

### Certainty of evidence assessment

The certainty of evidence was evaluated using the Grading of Recommendations, Assessment, Development, and Evaluation (GRADE) framework^14^. This was done only for the meta-analysis (or sensitivity analysis) reporting a statistically significant benefit of the intervention analysed.

### Meta-analyses

RCTs demonstrating a statistically significant reduction in the rate of POPF (*P* < 0.050) for the intervention under investigation were classified as positive. These potentially effective interventions were subsequently listed, and meta-analyses were performed only when at least three RCTs were available for a given intervention, regardless of their outcome. This approach, which considered only interventions shown to reduce POPF in at least one RCT, was chosen for pragmatic reasons, to prioritize interventions with preliminary evidence of efficacy. Three RCTs were considered the minimum required to undertake a meaningful meta-analysis^15^. Given that none of the meta-analyses included ten or more studies, formal assessment of publication bias (such funnel plot inspection or statistical tests for small-study effects) and meta-regression were not carried out.

### Statistical analysis

Descriptive statistics were used to summarize the extracted data. Continuous variables are presented as mean(standard deviation) or median (interquartile range, i.q.r.), as appropriate. Categorical variables are presented as numbers and percentages. The primary outcome (pooled incidence of POPF) was analysed using a meta-analysis of proportions. Individual study proportions were stabilized using a logit transformation (PLOGIT) and pooled using the inverse-variance method under a random-effects model. A random-effects approach was selected *a priori* because of the expected clinical and methodological heterogeneity across trials, including differences in surgical techniques, patient populations, perioperative management, and definitions of POPF over time.

Confidence intervals for pooled estimates were calculated using the Hartung–Knapp adjustment, which provides a more robust inference in the presence of heterogeneity and when the number of included studies is limited.

Data from the systematic review component are summarized descriptively in structured tables, whereas results of meta-analyses are presented graphically using forest plots displaying individual study estimates and pooled effects with corresponding 95% confidence intervals. The meta-analyses were performed for rate of POPF using R (R Foundation for Statistical Computing, Vienna, Austria). The *I*^2^ statistic was used to assess heterogeneity between studies. An *I*^2^ value greater than 50% was considered to indicate substantial heterogeneity. An inverse-variance random-effects model was used to calculate the pooled effect. Statistical significance was set at two-sided *P* < 0.050 for all analyses.

### Sensitivity analyses

Sensitivity analyses were conducted in accordance with the methodological recommendations outlined in the Cochrane Handbook for Systematic Reviews of Interventions^16^.

First, leave-one-out analyses were undertaken for all meta-analyses, irrespective of the statistical significance of the primary pooled estimate. Second, meta-analyses were repeated after excluding studies judged to be at high risk of bias according to the RoB-2 tool. Third, subgroup sensitivity analyses were carried out based on intervention characteristics (for example type of somatostatin analogue, type of surgery, type of sealant, type of cortisone, type of stent, type of anastomosis, type of surgical technique), whenever data were available.

### Amendments to registered protocol

The review was registered initially as a systematic evidence mapping study limited to RCTs using the 2016 ISGPS definition of POPF. During study conduct, eligibility was expanded to include both the 2005 and 2016 ISGPS definitions, and the study period was extended (2005–2025). To ensure consistency across definitions, the primary outcome was restricted to clinically relevant POPF (grade B/C). Additionally, quantitative syntheses were undertaken for interventions fulfilling the predefined inclusion criteria. These amendments were introduced to improve methodological consistency and comparability across studies.

## Results

### Study selection and publication details

**Fig. 1:**
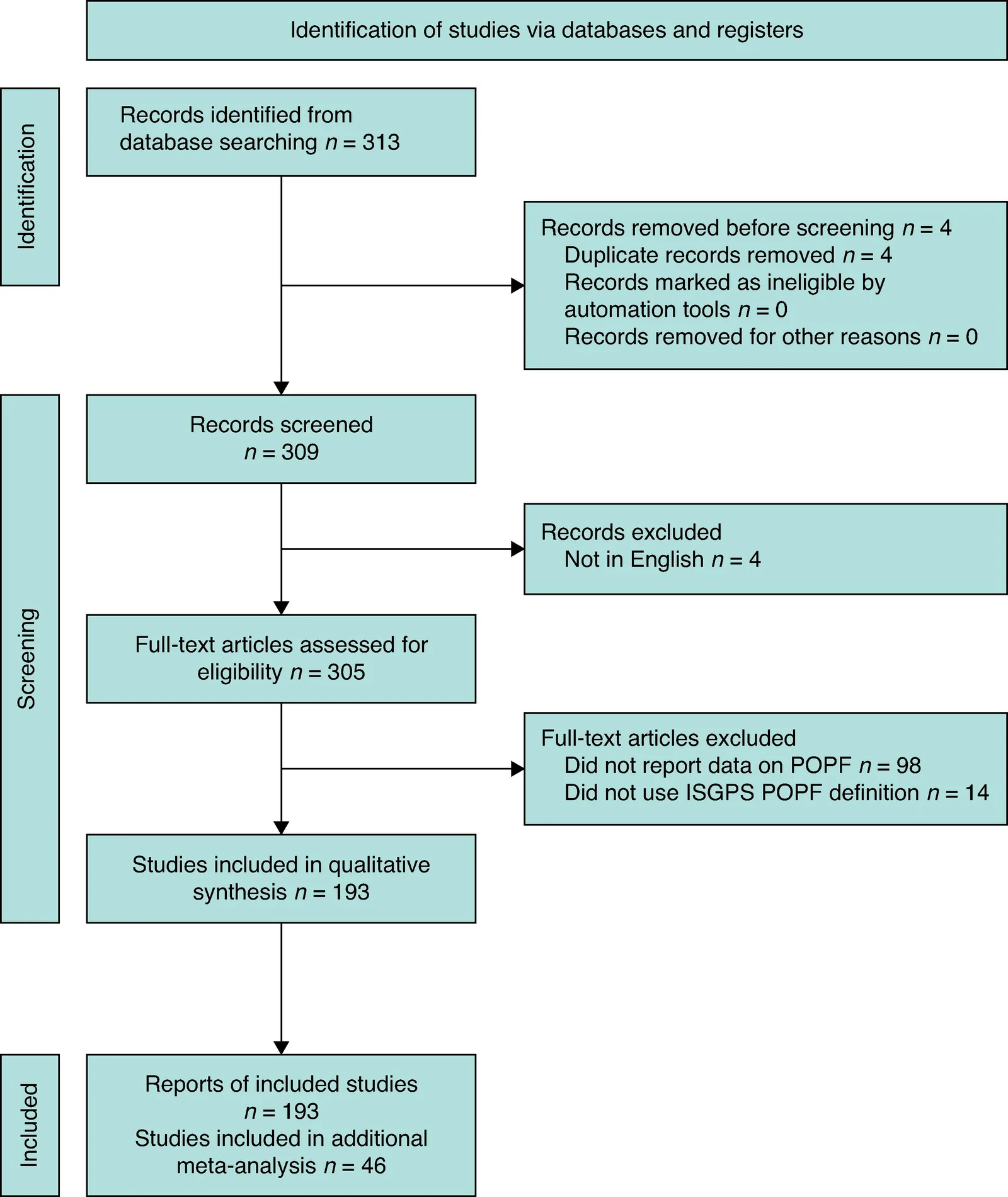
PRISMA flow chart showing selection of articles for review POPF, postoperative pancreatic fistula; ISGPS, International Study Group for Pancreatic Surgery.

The literature search identified 313 RCTs of which, after screening and eligibility evaluation, 193 (29 408 patients) were included in the present systematic review (*Fig. 1*). These 193 RCTs originated from 24 countries, and 70 (36.0%) were multicentre studies. Among the multicentre RCTs, 61 (87.0%) were performed in a single country, whereas 9 (13.0%) were international. The samples size ranged from 13 to 1748 patients (median 110, i.q.r. 100–123)^17,18^. Overall, 130 RCTs (68.0%) defined POPF according to the 2005 ISGPS definition and 63 (32.0%) according to the 2016 ISGPS definition. Among the 193 RCTs, 89 (46.1%) focused on pancreatoduodenectomy (PD), 29 (15.0%) on left pancreatectomy (LP), 69 (35.7%) on various perioperative interventions during any type of pancreatectomy, and 6 (3.0%) on other surgical aspects. Among the 193 included RCTs, 36 focused specifically on interventions for POPF, either preoperative, intraoperative, or postoperative (18.7%). Risk-of-bias assessments for trials reporting a statistically significant reduction in POPF and for studies included in the meta-analyses are presented in *Table S1*.

### Primary outcome

**Fig. 2:**
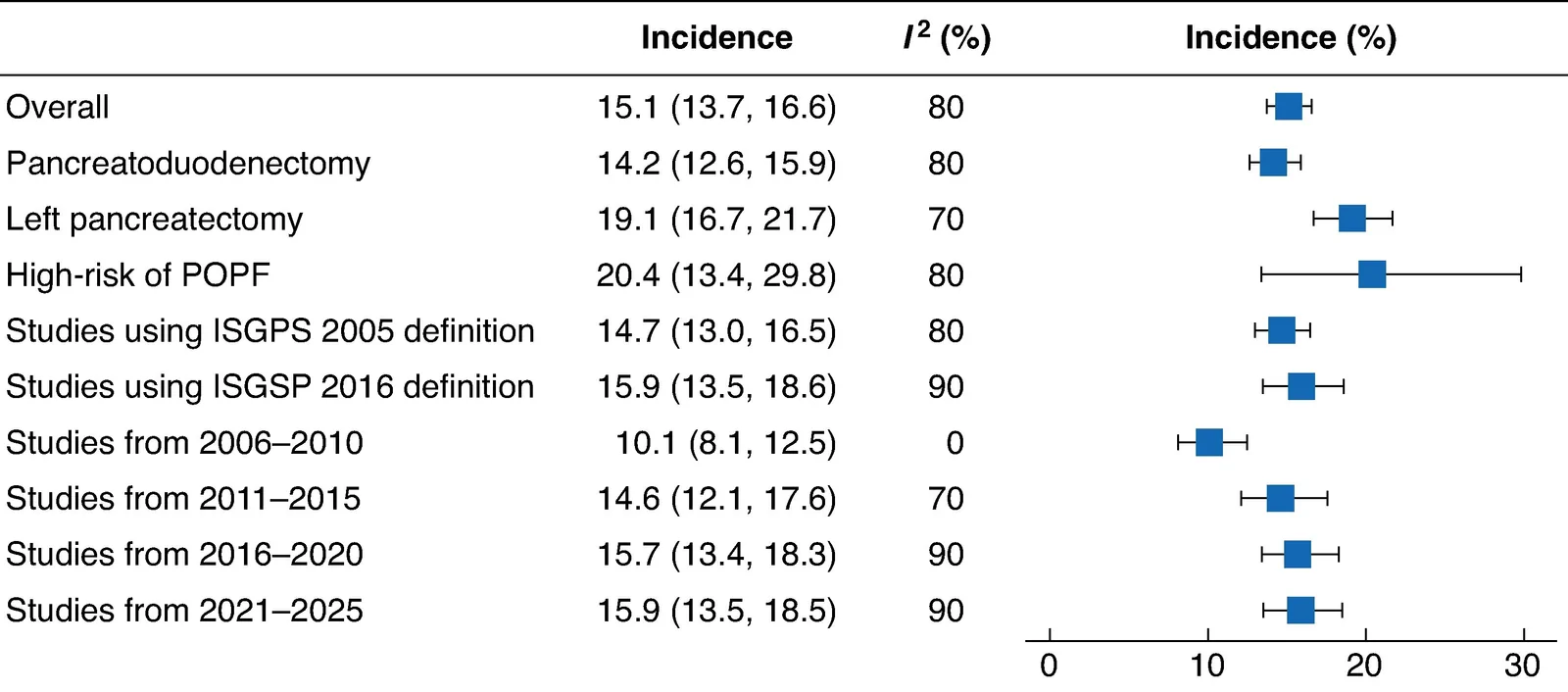
Pooled rates of POPF Values in parentheses are 95% confidence intervals. POPF, postoperative pancreatic fistula; ISGPS, International Study Group for Pancreatic Surgery.

Among the included RCTs, the pooled incidence of POPF was 15.1%. When stratified by procedure, the pooled rate was 14.2% after PD and 19.1% after LP (*Fig. 2*). In studies including exclusively high-risk patients, the pooled POPF rate was 20.4%.

Across 5-year intervals, POPF incidence increased over time, from 10.1% in 2005–2010 to 15.9% in 2021–2025. Rates were comparable before and after the 2016 ISGPS definition update (14.7% *versus* 15.9%).

### RCTs reporting on effective interventions

Overall, 24 RCTs^17,19–41^ that reported 18 interventions reduced the rate of POPF (*Table 1*). Of these, 11 (50.0%) assessed interventions related to the pancreatic anastomosis during PD, 2 focused on stump management in LP, 3 on drain management in both PD and LP, 2 on antibiotic prophylaxis in PD, 4 on pharmacological interventions in PD and LP, and 2 on other additional interventions in PD.

**Table 1:** RCTs reporting effective intervention for preventing POPF after partial pancreatectomy

Reference	Type of surgery	Primary aim	Sample size	POPF results	POPF rate (%)
**Stent *versus* no stent**					
Motoi *et al*.^19^ (2012)	PD	POPF rate after external stent *versus* no stent in duct-to-mucosa PJ anastomosis	93	POPF (stent 3 (6%) *versus* no stent 10 (22%); *P* = 0.040) POPF in non-dilated MPD (stent 2 (10%) *versus* no stent 8 (40%); *P* = 0.030)	14
**Duct-to-mucosa *versus* invagination PJ anastomosis**					
Senda *et al*.^20^ (2018)	PD	POPF rate after duct-to-mucosa *versus* invagination PJ anastomosis	120	POPF in soft pancreas (duct-to-mucosa 13 (42%) *versus* invagination 3 (10%); OR 0.16, 95% c.i. 0.04 to 0.65; *P* = 0.010)	16.7
Bai *et al*.^21^ (2016)	PD	POPF rate and grade after duct-to-mucosa *versus* invagination PJ anastomosis	132	POPF (duct-to-mucosa 2 (3.1%) *versus* invagination 12 (17.6%); *P* = 0.004)	10.6
Xu *et al*.^22^ (2015)	PD	POPF rate after duct-to-mucosa *versus* papillary-like PJ anastomosis	308	POPF (duct-to-mucosa 13 (8.5%) *versus* papillary-like 1 (0.6%); *P* = 0.034)	4.5
Berger *et al*.^23^ (2009)	PD	POPF rate after duct-to-mucosa *versus* invagination PJ anastomosis	197	Grade B POPF (duct-to-mucosa 14 (14%) *versus* invagination 5 (5%); *P* = 0.030)	12.2
**Other anastomotic techniques**					
Liu *et al*.^24^ (2021)	PD	POPF rate after robotic single-layer *versus* modified Blumgart PJ	182	POPF (single layer 6 (6.7%) *versus* modified Blumgart 11 (11.8%); *P* < 0.001) POPF in soft pancreas (single layer 3 (4.6%) *versus* modified Blumgart 10 (15.6%); *P* = 0.040)	9.3
Shao *et al*.^25^ (2019)	PD	POPF rate after PBRP PJ *versus* traditional PJ	70	POPF (PBRP 1 (2.9%) *versus* traditional 8 (22.8%); *P* = 0.028) POPF in pancreas soft (PBRP 1 (4.2%) *versus* traditional 7 (35%); *P* = 0.025)	12.9
**Sealant**					
Serradilla-Martín *et al*.^26^ (2023)	PD	POPF rate after PJ with 2 PEG-coated collagen-based haemostatic patches *versus* no patch	64	POPF (patch 1 (3%) *versus* no patch 8 (24%); *P* = 0.027; OR 0.12, 95% c.i. 0.00 to 0.73)	14.1
**PG *versus* PJ**					
Figueras *et al*.^27^ (2013)	PD	Incidence and severity of pancreatic ﬁstula after PD	123	POPF (PG 7 (11%) *versus* PJ 19 (33%); *P* < 0.001)	24.4
Topal *et al*.^28^ (2013)	PD	POPF rate after PG *versus* PJ end-to-side telescoped fashion anastomosis	329	POPF (PG 13 (8%) *versus* PJ 33 (10.9%); OR 2.86, 95% c.i. 1.38 to 6.17; *P* = 0.002)	14.0
**Other interventions**					
Yokoyama *et al*.^29^ (2014)	PD	Postoperative complications after replacement of externally drained pancreatic juice *versus* no replacement	46	POPF (replacement 2 (9.1%) *versus* no replacement 8 (33.3%); *P* = 0.046)	21.7
**Drainage**					
van Bodegraven *et al*.^30^ (2024)	LP	Clavien–Dindo grade ≥ III in drain *versus* no drain during LP	282	POPF (no drain 16 (12%) *versus* drain 39 (27%); *P* < 0.001)	19.5
Witzigmann *et al*.^31^ (2016)	PD	Overall reintervention rate after drain *versus* no drain	438	POPF (drain 24 (11.9%) *versus* no drain 11 (5.7%); *P* = 0.030) POPF (crossover 6 (15.0%) *versus* no drain 5 (3.3%); *P* = 0.004)	8.0
Dai *et al*.^32^ (2022)	PD	Clavien Dindo grade ≥ II after early *versus* routine drain removal	312	POPF in MI operations (early removal 0 (0%) *versus* routine removal 6 (17.1%); *P* = 0.009)	5.1
**Pharmaceutical co-treatment**					
D’Angelica *et al*.^33^ (2023)	PD	SSI after perioperative piperacillin–tazobactam *versus* cefoxitin	778	POPF (piperacillin–tazobactam 48 (12.7%) *versus* cefoxitin 76 (19.0%); OR 0.62, 95% c.i. 0.40 to 0.96; *P* = 0.030)	15.9
Yamamoto *et al*.^34^ (2018)	PD	Rate of infectious complications after 1 *versus* 5 days of cefozopran as antimicrobial prophylaxis	82	POPF (1 day 3 (8%) *versus* 5 days 10 (24%); *P* = 0.038)	15.9
Zhang *et al*.^35^ (2016)	PD	28-day postoperative survival after perioperative ulinastatin *versus* no ulinastatin	106	POPF (ulinastatin 3 (7%) *versus* no ulinastatin 12 (24%); *P* = 0.045) POPF in MPD < 3 (ulinastatin 2 (11%) *versus* no ulinastatin 8 (32%); *P* = 0.022)	14.2
Cao *et al*.^36^ (2021)	PD	POPF rate after somatostatin *versus* no somatostatin in intermediate-risk FRS	205	POPF (somatostatin 13 (13%) *versus* standard 25 (25%); *P* = 0.032)	18.5
Allen *et al*.^37^ (2014)	PD, LP	Grade ≥ 3 POPF, leak, or abscess as defined by MSKCC Surgical Secondary Events system after perioperative pasireotide *versus* placebo	300	POPF (pasireotide 12 (7.9%) *versus* no pasireotide 25 (16.9%); *P* = 0.020)	12.3
Antila *et al*.^17^ (2019)	LP	Overall complication rate after perioperative hydrocortisone *versus* placebo	31	POPF (hydrocortisone 1 (6%) *versus* placebo 6 (43%); *P* = 0.028)	22.6
**Nutrition**					
Perinel *et al*.^38^ (2016)	PD	Postoperative complication after enteral *versus* total parenteral nutrition	204	POPF (enteral 30 (29.4%) *versus* total parenteral 14 (13.9); *P* = 0.007)	21.6
**Sealants**					
Park *et al*.^39^ (2020)	PD, LP	POPF rate after flowable thrombin–collagen-based haemostatic matrix *versus* thrombin-coated collagen patch	25	POPF (flowable haemostatic matrix 1 (8.3%) *versus* patch 6 (46.2%); *P* = 0.027) Grade B POPF (flowable haemostatic matrix 1 (8.3%) *versus* patch 5 (38.5%); *P* = 0.018) Grade C POPF (flowable haemostatic matrix 0 (0%) *versus* patch 1 (7.7%); *P* = 0.018)	28
Jang *et al*.^40^ (2017)	LP	POPF rate after polyglycolic acid mesh *versus* no-mesh	97	POPF (mesh 5 (11.4%) *versus* no mesh 15 (28.3%); *P* = 0.040)	20.6
Hamilton *et al*.^41^ (2012)	LP	POPF rate after stapled transection with mesh buttress device reinforcement *versus* stapled transection alone	55	POPF (mesh 1 (1.9%) *versus* no mesh 11 (20%); *P* = 0.007)	21.8

RCT, randomized clinical trial; POPF, postoperative pancreatic fistula; PD, pancreatoduodenectomy; PJ, pancreatojejunostomy; MPD, main pancreatic duct; OR, odds ratio; c.i., confidence interval; PBRP, polytetrafluoroethylene suture buttress-reinforced pancreatojejunostomy; PEG, polyethylene glycol; PG, pancreatogastrostomy; LP, left pancreatectomy; MI, minimally-invasive; SSI, surgical site infection; FRS, fistula risk score; MSKCC, Memorial Sloan Kettering Cancer Center.

#### Pancreatic anastomosis in PD

External stent placement was reported to reduce the incidence of POPF after PD in one RCT^19^. A duct-to-mucosa pancreatojejunostomy (PJ) during PD reduced POPF compared with invagination PJ in one RCT, whereas three trials reported the opposite effect^20–23^. Polytetrafluoroethylene suture buttress-reinforced suture PJ during PD reduced POPF compared with a standard (non-reinforced) PJ in one trial^25^. A single-layer continuous suturing PJ reduced POPF in robot-assisted PD compared with a modified Blumgart PJ, both in the overall PD population and a high-risk subgroup with soft pancreatic parenchyma in one RCT^24^. Pancreatogastrostomy (PG) was effective in reducing POPF during PD compared with PJ in two RCTs^27,28^. A polyethylene glycol-coated patch reduced POPF during PD in one RCT, whereas a flowable collagen-based haemostatic (Hemopatch^™^ Sealing Hemostat, Baxter Aktiengesellschaft, Vienna, Austria) was found to be effective compared with a thrombin-coated L-dopa-containing collagen patch (CollaSeal^®^, Dalim Tissen Co. Ltd., Seul, Korea) in another trial^26,39^.

#### Stump management in LP

Seamguard^®^ mesh-reinforced pancreatic stapling during LP reduced POPF in one RCT as did polyglycolic acid mesh-reinforced pancreatic stapling during LP compared with stapling alone in one trial^40,41^.

#### Drain management in PD and LP

A no-drain policy following PD and LP, respectively, reduced POPF in two RCTs^30,31^. Additionally, early drain removal (on postoperative day (POD) 3) after PD reduced POPF compared with standard drain removal (on/beyond POD 5) in one trial^32^.

#### Antibiotic prophylaxis in PD

Piperacillin–tazobactam prophylaxis during PD reduced POPF compared with cefoxitin in one RCT^33^. Additionally, 1 day of antibiotic prophylaxis reduced POPF compared with 5 days of prophylaxis after PD following biliary drainage in another trial^34^.

#### Pharmacological interventions in PD and LP

Postoperative ulinastatin after PD reduced POPF in one RCT, whereas use of a perioperative/postoperative somatostatin analogue after PD and LP reduced POPF in two trials^35–37^. Furthermore, perioperative/postoperative hydrocortisone in LP reduced POPF in one RCT^17^.

#### Other additional interventions in PD

Following PD, total parental nutrition reduced POPF compared with early enteral nutrition in one RCT^38^. Enteral reinfusion of externally drained pancreatic juice was found to be effective in reducing POPF after PD in another trial^29^.

### Secondary outcomes

Risk factors for POPF were reported in 28 RCTs^19,21–23,27,35,39,40,42–61^ (*Table S2*). For interventions included in the meta-analyses, additional postoperative outcomes were collected, including DGE (grade B/C), PPH (grade B/C), LOS, and mortality (*Table S3*).

### RCTs in high-risk patients

Among 193 RCTs, 29 focused on patients at high-risk of POPF in PD and LP. Among these, 11 focused on patients at high risk of POPF in the primary outcome, whereas the remaining 18 included these as subgroup analyses.

### RCTs combining interventions

All RCTs were focused on a single intervention and no trial combined simultaneous interventions.

### RCTs in ongoing POPF

Four RCTs focused on direct treatment of an ongoing POPF. In 2018, a single-centre RCT by Takeda *et al*.^62^ investigated the role of coagulation factor XIII administration after POD 7 in patients with < 70.0% of plasma factor XIII level after both PD and LP, and reported no improvement in the primary endpoint (duration of drain placement from POD 7) and secondary endpoint (duration of drain placement from operation) between the intervention and the control groups. In 2019, Wu *et al*.^63^ published a trial demonstrating the non-inferiority of oral feeding compared with enteral nutrition in achieving 30-day fistula closure among patients with POPF after PD. In 2015, two multicentre RCTs by Fujii *et al*.^55,64^ compared oral nutrition with parenteral nutrition in patients with a biochemical leak after PD and LP, respectively. Progression from biochemical leak to POPF and duration of drain placement did not differ between the two groups.

### Meta-analyses of RCTs

**Fig. 3:**
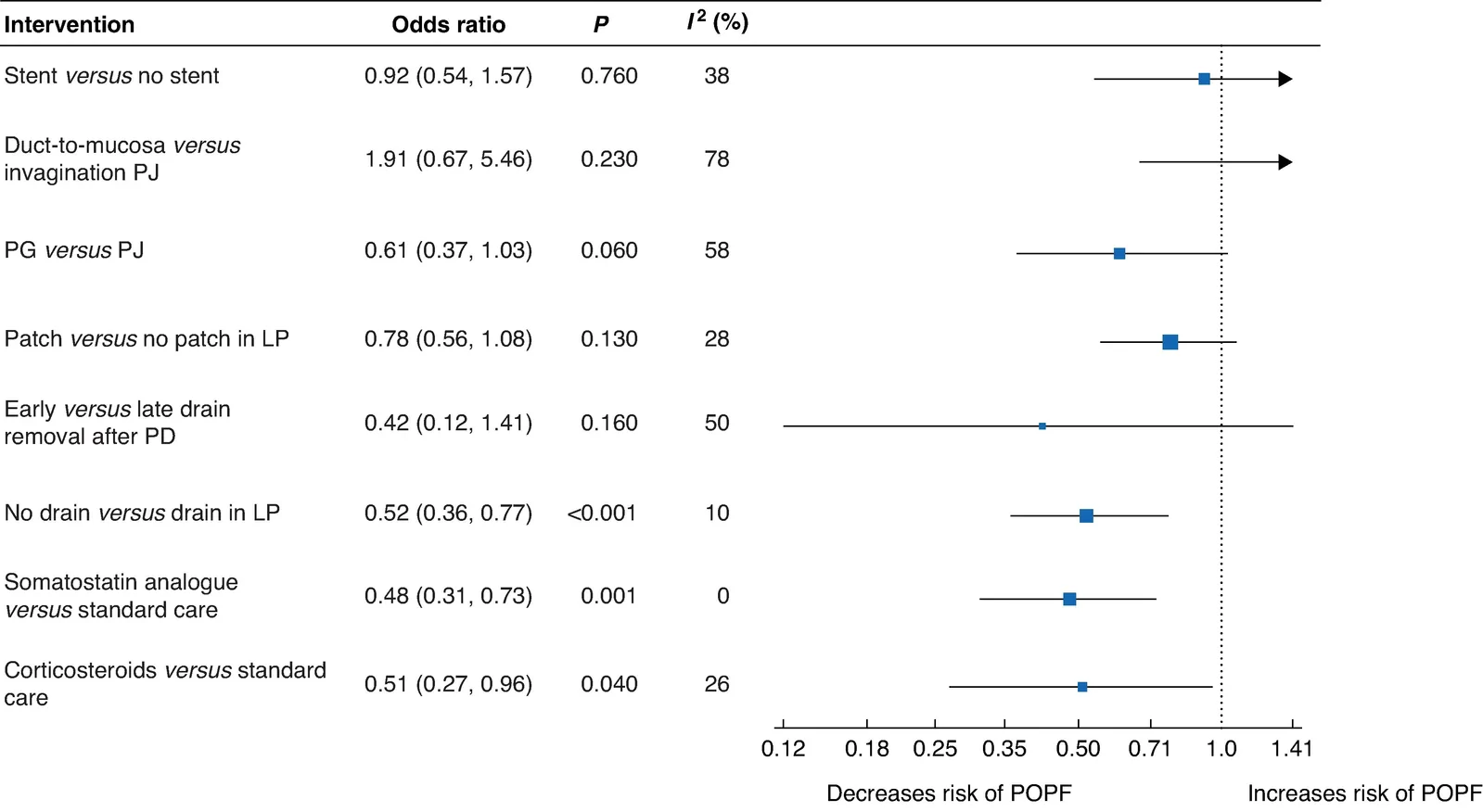
Forest plot of the eight meta-analyses showing the effects of various interventions on the risk of POPF Odds ratios are shown with 95% confidence intervals. POPF, postoperative pancreatic fistula; PJ, pancreatojejunostomy; PG, pancreatogastrostomy; LP, left pancreatectomy; PD, pancreatoduodenectomy.

Of the 18 effective interventions identified in the systematic review, 8 were eligible for meta-analysis as at least 1 RCT was positive and, overall, 3 trials of that intervention were available. Overall, 46 RCTs were included in the 8 meta-analyses regarding these 8 interventions: stent *versus* no stent in PD, duct-to-mucosa *versus* invagination PJ in PD, PG *versus* PJ, sealant use after LP, early *versus* late drain removal after PD, drain *versus* no drain policy in LP, use of perioperative somatostatin analogues, and perioperative corticosteroids administration (*Fig. 3*).

#### Pancreatic duct stent *versus* no stent

**Fig. 4:**
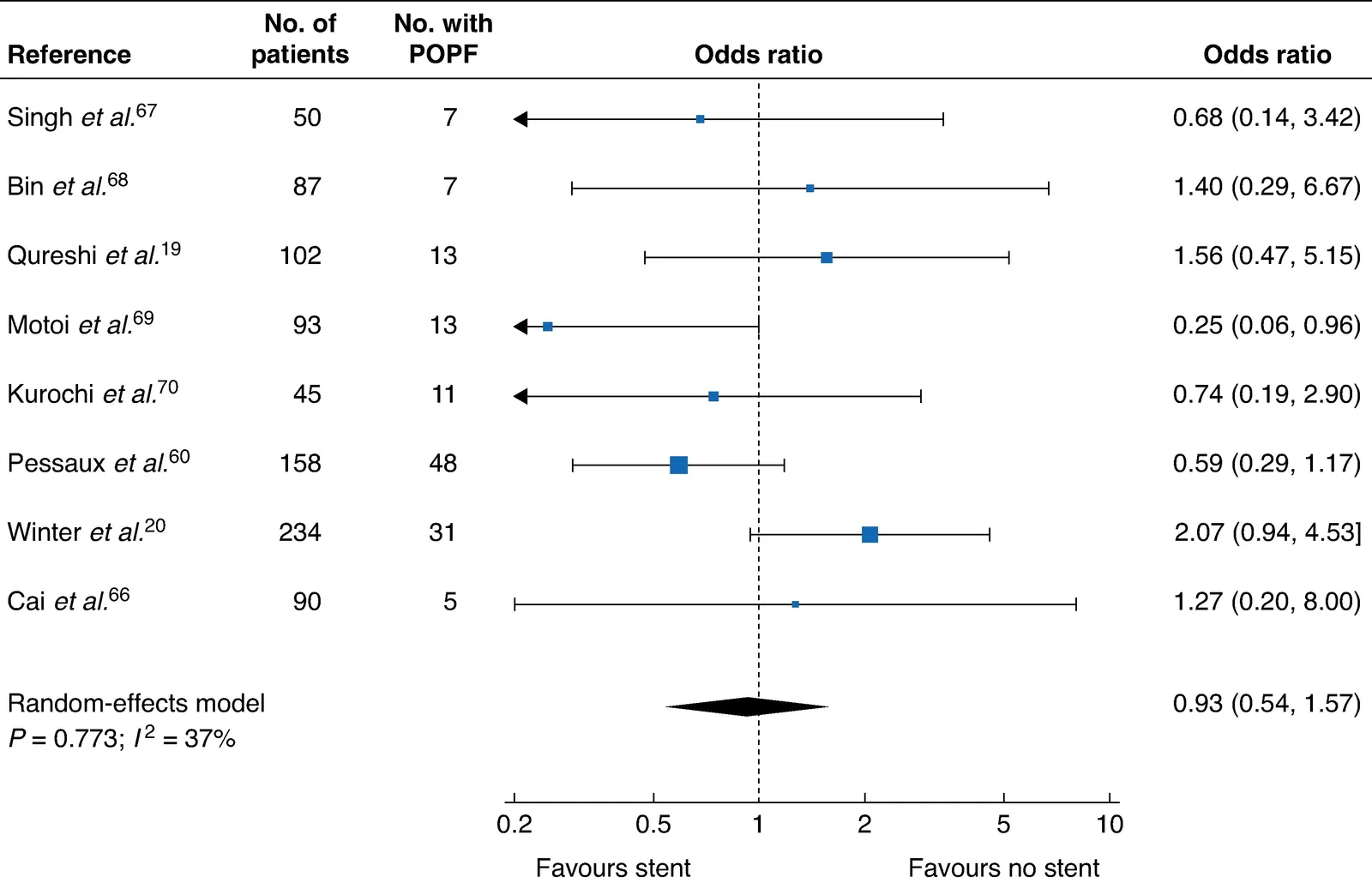
Forest plot showing effect of stent *versus* no stent on POPF rate Odds ratios are shown with 95% confidence intervals. POPF, postoperative pancreatic fistula.

Eight RCTs^19,60,65–70^ focused on pancreatic duct stenting in PD *versus* no stenting, including 769 patients. Among these, six RCTs (85.7%) focused on PJ with a stent and one on both PJ and PG with stent placement^70^. Stent placement did not result in a significantly reduced POPF risk (stent *versus* no stent: odds ratio (OR) 0.93, 95% confidence interval (c.i.) 0.54 to 1.57; *P* = 0.773, *I*^2^ = 37%) (*Fig. 4*).

#### Duct-to-mucosa *versus* invagination pancreatic anastomosis

**Fig. 5:**
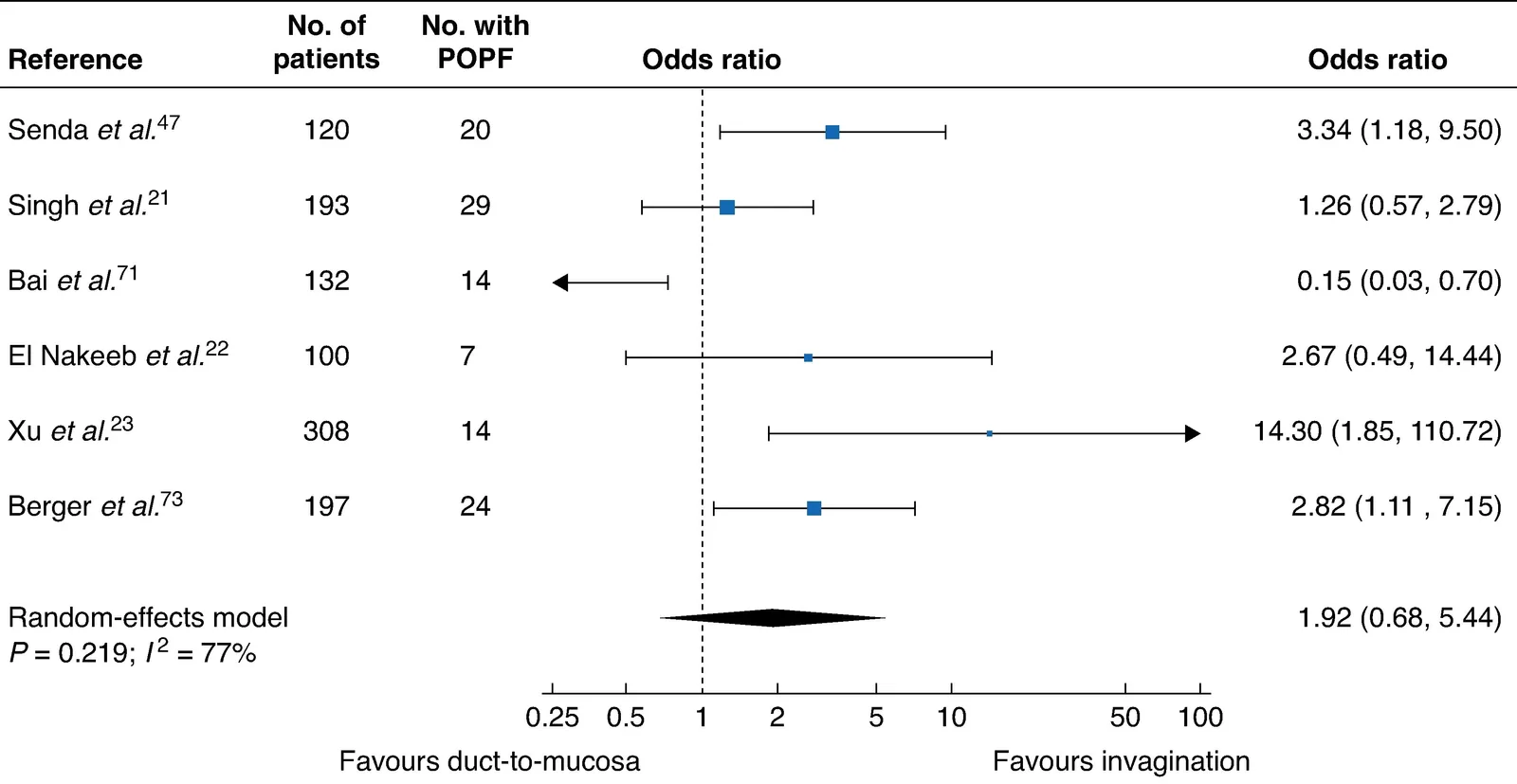
Forest plot showing effect of duct-to-mucosa *versus* invagination pancreatojejunostomy on POPF rate Odds ratios are shown with 95% confidence intervals. POPF, postoperative pancreatic fistula.

Six RCTs^20–23,47,71^ compared duct-to-mucosa PJ during PD with an invagination technique, including 1050 patients. Duct-to-mucosa PJ did not significantly reduce the risk of POPF (duct-to-mucosa *versus* invagination: OR 1.92, 95% c.i. 0.68 to 5.44; *P* = 0.219, *I*^2^ = 77%) (*Fig. 5*).

#### PG *versus* PJ pancreatic anastomosis

**Fig. 6:**
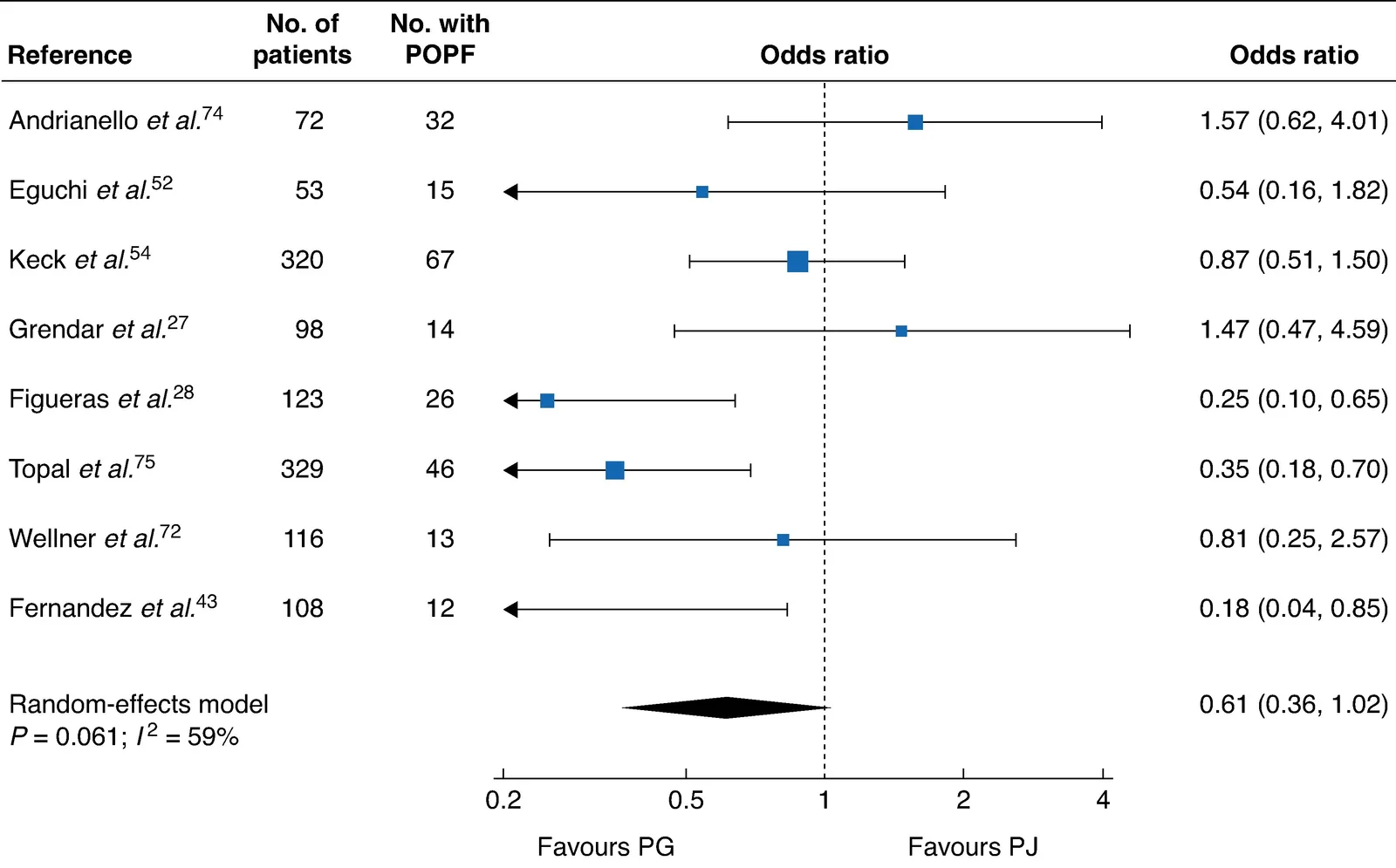
Forest plot showing effect of pancreatojejunostomy *versus* pancreatogastrostomy on POPF rate Odds ratios are shown with 95% confidence intervals. POPF, postoperative pancreatic fistula.

Eight RCTs^27,28,52,54,72–75^ compared PG and PJ anastomosis, including 1219 patients. The PG anastomosis was associated with a lower POPF rate, but the difference was not statistically significant (PG *versus* PJ: OR 0.61, 95% c.i. 0.36 to 1.02; *P* = 0.061, *I*^2^ = 59%) (*Fig. 6*).

#### Sealant on pancreatic stump in LP

**Fig. 7:**
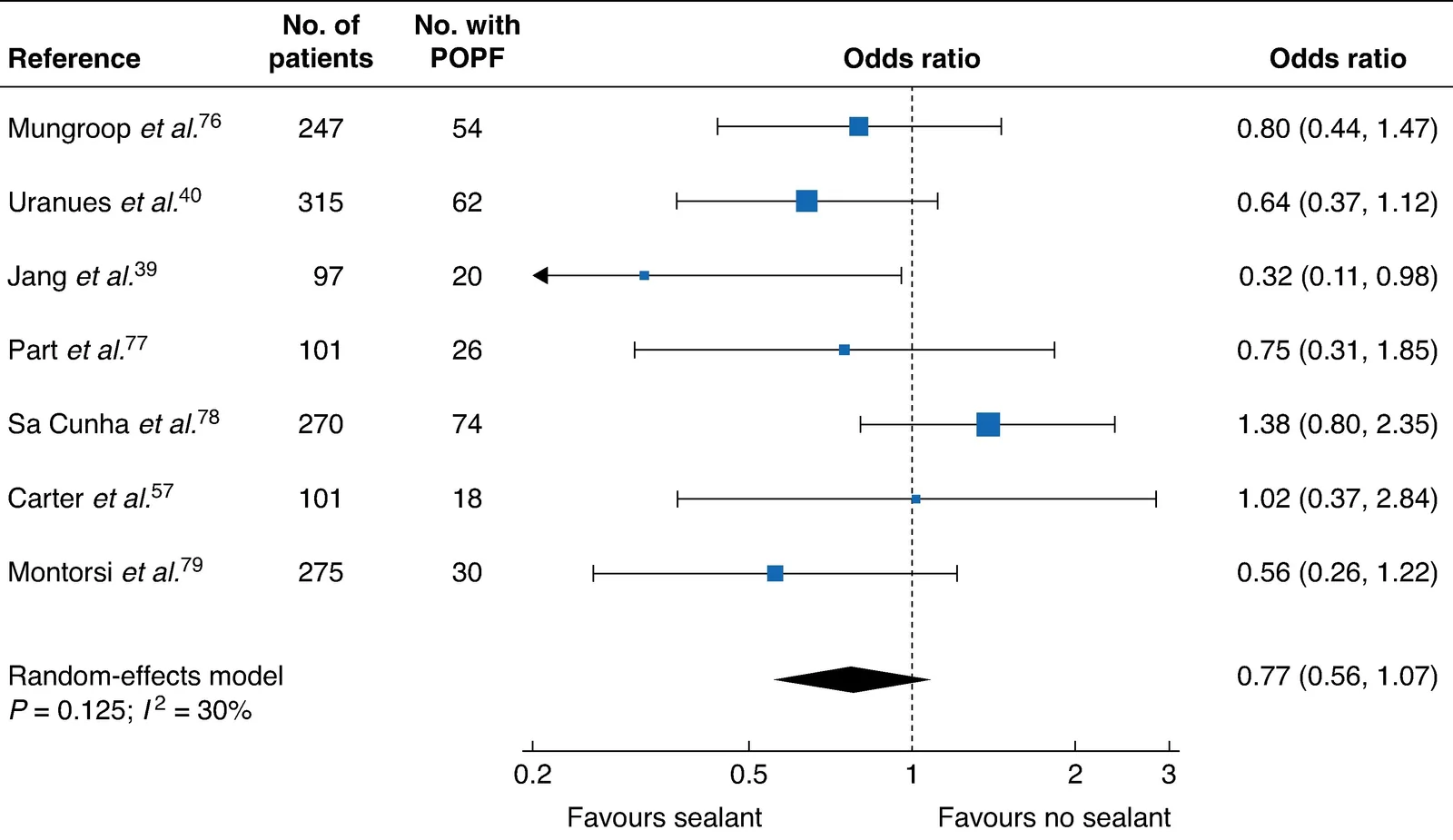
Forest plot showing effect of sealant on pancreatic stump on POPF rate in left pancreatectomy Odds ratios are shown with 95% confidence intervals. POPF, postoperative pancreatic fistula.

Seven RCTs^39,40,43,57,76–78^ analysed the efficacy of using a patch to cover the pancreatic remnant after LP, including 1406 patients. Patch placement did not reduce POPF after LP (patch *versus* no patch: OR 0.77, 95% c.i. 0.56 to 1.07; *P* = 0.125, *I*^2^ = 30%) (*Fig. 7*).

#### Early *versus* late drain removal after PD

**Fig. 8:**
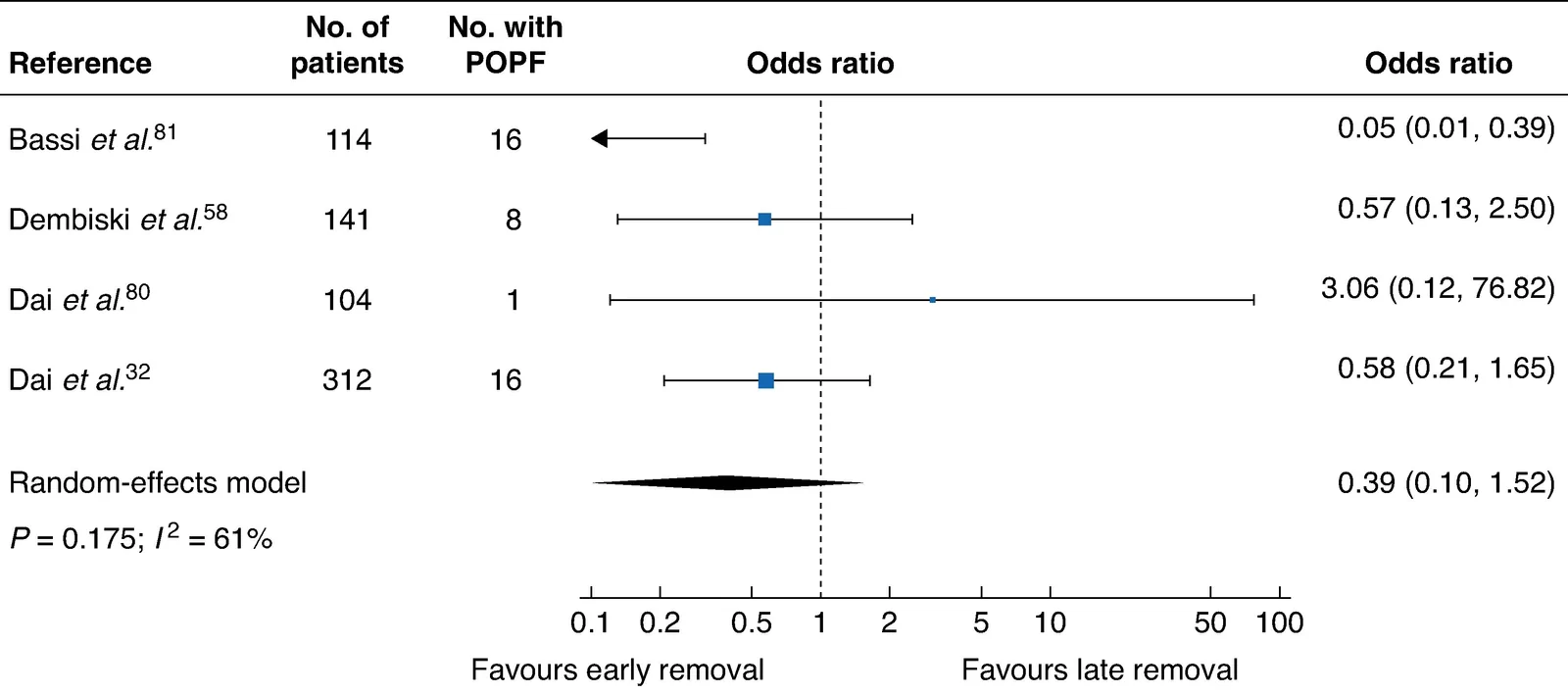
Forest plot showing effect of early *versus* late drain removal after pancreatoduodenectomy on POPF rate Odds ratios are shown with 95% confidence intervals. POPF, postoperative pancreatic fistula.

Four RCTs^32,58,79,80^ analysed the efficacy of early (POD 3) compared with late (> POD 3) drain removal following PD, including 671 patients. Early drain removal did not significantly reduce POPF (early *versus* late: OR 0.39, 95% c.i. 0.10 to 1.52; *P* = 0.175, *I*^2^ = 61%) (*Fig. 8*).

#### Drain *versus* no drain placement in LP

**Fig. 9:**
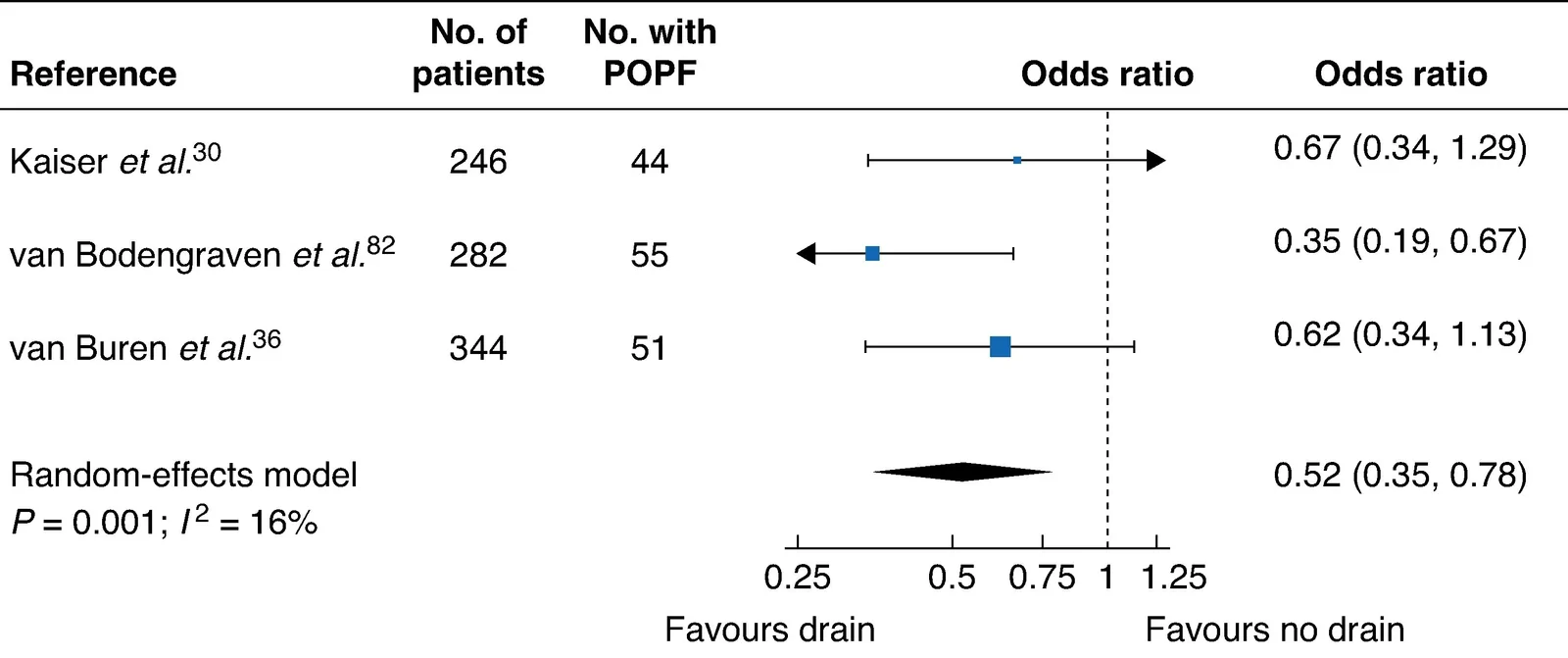
Forest plot showing effect of drain *versus* no drain placement in left pancreatectomy Odds ratios are shown with 95% confidence intervals. POPF, postoperative pancreatic fistula.

Three RCTs^30,81,82^, including 872 patients, investigated the role of abdominal drainage in LP. Omission of drains in LP was associated with a statistically significant reduction in POPF risk (no drain *versus* drain: OR 0.52, 95% c.i. 0.35 to 0.78; *P* = 0.001, *I*^2^ = 16%) (*Fig. 9*).

#### Perioperative somatostatin analogues

**Fig. 10:**
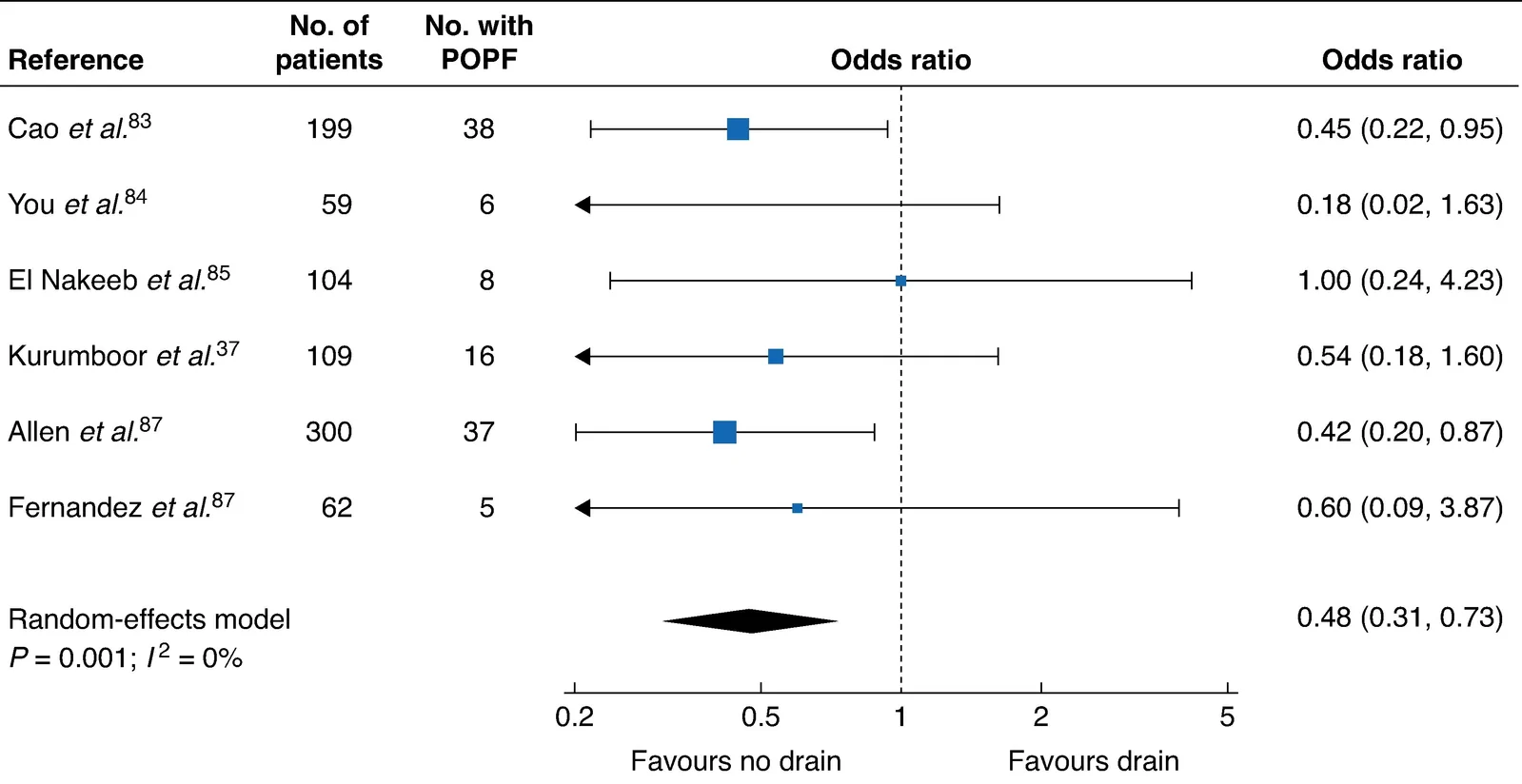
Forest plot showing impact of perioperative somatostatin analogues on POPF rate Odds ratios are shown with 95% confidence intervals. POPF, postoperative pancreatic fistula.

Six RCTs^36,37,83–86^ analysed the efficacy of somatostatin analogue administration in both PD and LP, including 833 patients. Administration of somatostatin analogues significantly reduced POPF (somatostatin analogue *versus* standard care: OR 0.48, 95% c.i. 0.31 to 0.73; *P* = 0.001, *I*^2^ = 0%) (*Fig. 10*).

#### Perioperative corticosteroids

**Fig. 11:**
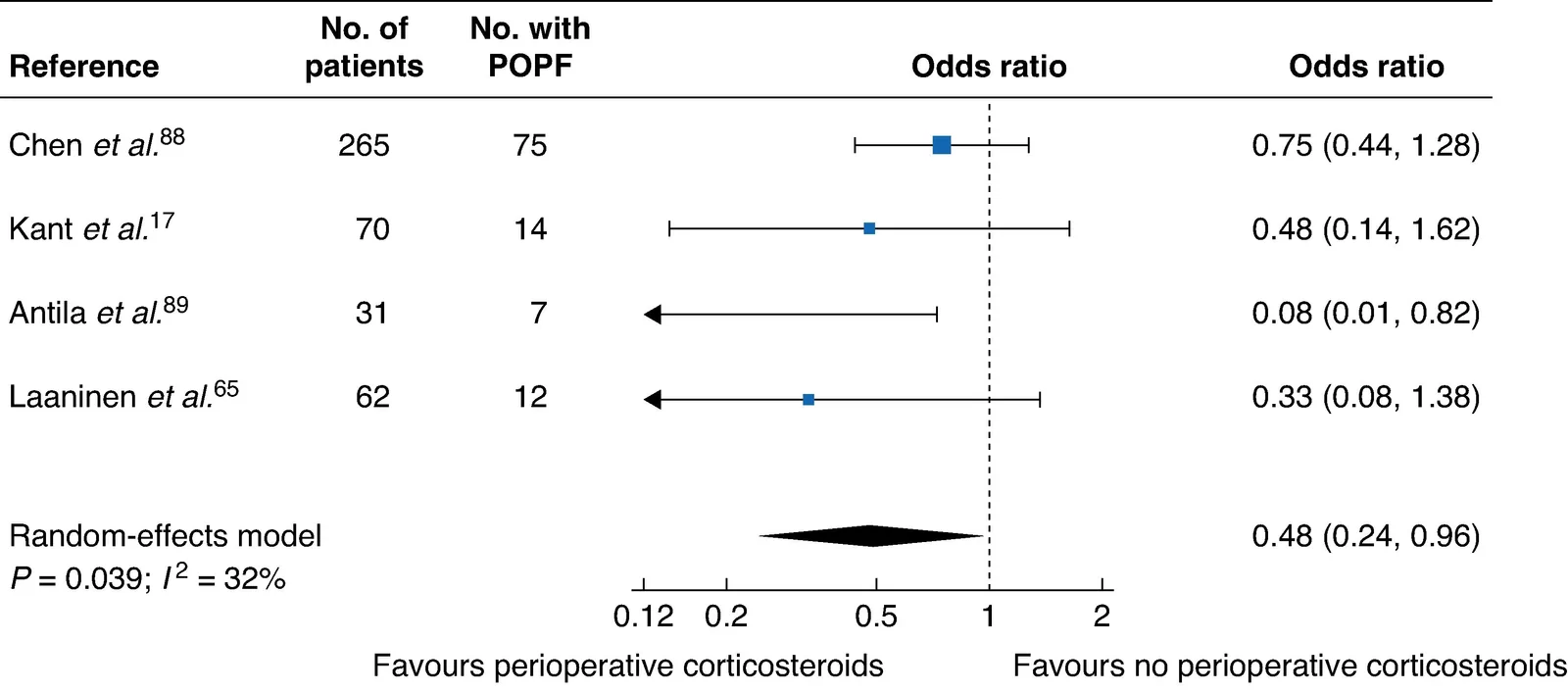
Forest plot showing impact of perioperative corticosteroids on POPF rate Odds ratios are shown with 95% confidence intervals. POPF, postoperative pancreatic fistula.

Four RCTs^17,87–89^, with a total of 428 patients, analysed the role of perioperative corticosteroid administration in PD and LP. Perioperative corticosteroids significantly reduced POPF (corticosteroids *versus* standard care: OR 0.48, 95% c.i. 0.24 to 0.96; *P* = 0.039, *I*^2^ = 32%) (*Fig. 11*).

### Sensitivity analyses

Several sensitivity analyses were conducted to assess the robustness of the findings.

#### Leave-one-out analyses

Leave-one-out analyses were performed for all eight meta-analyses to assess the influence of individual studies on the pooled estimates (*Figs S1* and *S2*). In several analyses, exclusion of individual trials led to changes in statistical significance; however, the direction of the effect estimates remained unchanged.

For drain omission after distal pancreatectomy and perioperative somatostatin analogues, the pooled estimates remained statistically significant in all iterations, confirming the robustness of these findings.

For perioperative corticosteroids, the pooled estimate was more sensitive to the exclusion of individual trials, with loss of statistical significance after removal of some studies, reflecting the relatively small number of available RCTs.

For the remaining interventions (duct-to-mucosa *versus* invagination PJ, early *versus* late drain removal, PG *versus* PJ, pancreatic stump sealants, and pancreatic duct stenting), leave-one-out analyses did not materially change the overall interpretation of the results.

#### Leave multiple studies out: RCTs with high risk of bias excluded

Sensitivity analyses excluding studies with a high risk of bias were undertaken for all meta-analyses with at least one such RCT (somatostatin analogues, pancreatic duct stent *versus* no stent, PG *versus* PJ, sealant in LP). No additional analyses were performed for early *versus* late drain removal as there were fewer than three RCTs after exclusion of trials at high risk of bias.

Exclusion of the RCTs with a high risk of bias from meta-analyses of the impact of somatostatin analogues confirmed the positive protective effect of somatostatin analogues (OR 0.49, 95% c.i. 0.31 to 0.76; *P* = 0.002, *I*^2^ = 0%) (*Fig. S3*).

Regarding pancreatic duct stenting, when only RCTs with a low–moderate risk of bias were included, the pooled effect size became significant for pancreatic duct stenting (OR 0.56, 0.32 to 0.96; *P* = 0.036), with no observed heterogeneity (*I*^2^ = 0% *versus* 38.0% when all studies were included) (*Fig. S4*).

Excluding trials at high risk from the meta-analyses of PG *versus* PJ revealed a pooled OR of 0.66 (0.37 to 1.20; *P* = 0.172), with persistent heterogeneity (*I*^2^ = 65% *versus* 58% when all studies were included). Although the direction of the effect remained favourable, statistical significance was not achieved (*Fig. S5*).

Regarding the use of sealant after LP, the meta-analyses showed a positive protective effect of the intervention after the exclusion of all three RCTs at high risk of bias (OR 0.66, 0.46 to 0.94; *P* = 0.023, *I*^2^ = 0%) (*Fig. S6*).

#### Leave multiple studies out: subgroup analyses

For the meta-analysis regarding perioperative somatostatin analogues, when only patients undergoing PD were included, administration of somatostatin analogues still significantly reduced POPF (OR 0.51, 95% c.i. 0.30 to 0.86; *P* = 0.011) (*Fig. S7*), with heterogeneity remaining at 0%. When only studies with octreotide were included, the reduction in POPF rate was not statistically significant (OR 0.57, 0.27 to 1.20; *P* = 0.138), while *I*^2^ remained at 0% (*Fig. S8*).

For the meta-analysis regarding perioperative corticosteroid administration, when only RCTs focusing on PD were included, the direction of the effect remained favourable, although statistical significance was not achieved (OR 0.64, 95% c.i. 0.40 to 1.02; *P* = 0.061, *I*^2^ = 0%) (*Fig. S9*). Additionally, when only studies with hydrocortisone were included, the reduction of POPF remained statistically significant (OR 0.33, 0.14 to 0.78; *P* = 0.011, *I*^2^ = 0%) (*Fig. S10*).

For the meta-analysis regarding pancreatic stump sealants in LP, when only the four RCTs using Tachosil^®^ were analysed, no significant reduction in POPF was found (OR 0.88, 0.58 to 1.33; *P* = 0.554) (*Fig. S11*) and heterogeneity remained substantial (*I*^2^ = 32% *versus* 28% in main analysis).

For the meta-analysis regarding stent *versus* no stent in PD, excluding the study that included both PJ and PG did not result in a reduction in rate of POPF (stent *versus* no stent: OR 1.05, 0.57 to 1.91; *P* = 0.886, *I*^2^ = 33.0%) (*Fig. S12*). Additionally, two subgroup analyses including the four RCTs that used only external stents or the three trials that used internal stents also failed to demonstrate a significant benefit of stenting (external stent: OR 0.70, 0.41 to 1.19, *P* = 0.190; internal stent: OR 1.61, 0.83 to 3.12, *P* = 0.155) (*Figs S13* and *S14*), with a substantial decrease in heterogeneity (internal stent: *I*^2^ = 0% *versus* 38.0%; external stent: *I*^2^ = 11.0% *versus* 38.0% in main analysis).

In the meta-analysis of PG *versus* PJ, exclusion of the only study that used pancreatic stenting together with PG and PJ^73^ resulted in a statistically significant pooled effect (OR 0.54, 0.32 to 0.89; *P* = 0.016), with persistent heterogeneity (*I*^2^ = 50.0% *versus* 58.0% in main analysis) (*Fig. S15*).

### Certainty of evidence

The certainty of evidence was assessed using the GRADE approach (*Table 2*). High-certainty evidence was observed only for drain omission after LP, indicating that avoiding routine use of prophylactic drains reduces the risk of POPF. For somatostatin analogues, the certainty of evidence was rated as moderate owing to risk of bias, suggesting a potential reduction in POPF after pancreatic resection, including in analyses restricted to PD.

**Table 2:** Certainty of evidence (GRADE)

Intervention	Analysis	Certainty of evidence (GRADE)	Reason for downgrading	Interpretative statement
Drain in LP	All included studies	High	No downgrading	High-certainty evidence suggests that omitting prophylactic drains after LP reduces risk of POPF compared with routine drain placement
Somatostatin analogues	All included studies	Moderate	Risk of bias	Moderate-certainty evidence suggests that perioperative somatostatin analogues may reduce risk of POPF after pancreatic resection
	Only in PD	Moderate	Risk of bias	Moderate-certainty evidence suggests a reduction in risk of POPF with perioperative somatostatin analogues in patients undergoing PD
Corticosteroids	All included studies	Low	Indirectness and imprecision	Low-certainty evidence suggests that perioperative corticosteroids may reduce risk of POPF; however, estimate is uncertain and further well designed trials may substantially change observed effect
	Only studies using hydrocortisone (no dexamethasone)	Low	Indirectness and imprecision	Low-certainty evidence suggests that perioperative hydrocortisone may reduce risk of POPF; however, estimate remains uncertain and further well designed trials are needed
PG *versus* PJ	After exclusion of study with distinct technique^73^	Low	Risk of bias and inconsistency	Low-certainty evidence suggests that PG may reduce risk of POPF compared with PJ; however, estimate is uncertain owing to heterogeneity and study limitations
Pancreatic duct stent *versus* no stent	After exclusion of RCTs with high risk of bias	Moderate	Indirectness	Low-certainty evidence suggests that pancreatic duct stenting may reduce risk of POPF after PD; however, estimate is uncertain owing to clinical heterogeneity and study limitations
Sealant after LP	After exclusion of RCTs at high risk of bias	Moderate	Indirectness	Moderate-certainty evidence suggests that use of a patch or sealant during pancreatic stump closure may reduce risk of clinically relevant POPF after distal pancreatectomy

GRADE, Grading of Recommendations, Assessment, Development, and Evaluation; LP, left pancreatectomy; POPF, postoperative pancreatic fistula; PD, pancreatoduodenectomy; PG, pancreatogastrostomy; PJ, pancreatojejunostomy; RCT, randomized clinical trial.

For perioperative corticosteroids, the certainty of evidence was low when all trials were considered because of indirectness and imprecision, but it increased to moderate in an analysis restricted to PD, although estimates remained imprecise.

Low-certainty evidence suggested that PG may reduce POPF compared with PJ, with downgrading driven by risk of bias and inconsistency.

For pancreatic duct stenting, the certainty of evidence was moderate after exclusion of trials with a high risk of bias but downgraded for indirectness owing to heterogeneity in stent types and surgical techniques.

Finally, evidence of moderate certainty suggested that the use of sealants or patches during pancreatic stump closure after LP may reduce POPF, although variation in sealant materials led to downgrading for indirectness.

## Discussion

This systematic review that included only RCTs following partial pancreatectomy identified 18 interventions from 24 RCTs as being effective in preventing POPF. However, following meta-analyses (restricted to interventions with ≥ 3 RCTs available) and sensitivity analyses, it became evident that the current evidence is immature. Only three interventions remained effective, with varying certainty of evidence according to the GRADE approach: no drain in LP (high certainty), perioperative somatostatin analogues (moderate certainty), and perioperative corticosteroids (low certainty). Additionally, for three interventions (PG *versus* PJ, pancreatic duct stenting, and sealant after LP), despite negative meta-analyses, high consistency (> 50%) and changes in results after sensitivity analyses indicated a high likelihood that future RCTs would affect the outcome. Only a minority of RCTs (11) focused on patients with a high risk of POPF, and no RCT combined interventions simultaneously.

This is the first meta-analysis to include only RCTs evaluating POPF after all types of partial pancreatectomy. As such, the results of this study cannot be compared fully with earlier research. In the PD setting, a 2022 meta-analysis of RCTs by the PARANOIA study group^90^ focused on POPF prevention. Among the 22 interventions assessed, only external pancreatic duct drainage was identified as effective (OR 0.40, 95% c.i. 0.20 to 0.80; *I*^2^ = 17%). This finding was not confirmed in the present analysis, which included 5 RCTs (compared with 3 trials in the PARANOIA study) and excluded the 2007 RCT by Poon *et al*.^59^, as it did not apply the ISGPS definition of POPF. Notably, the PARANOIA group also reported that two-thirds of the RCTs (19 of 25) included in the analysis of POPF were underpowered.

Regarding the omission of abdominal drains in LP, the present meta-analysis is the first to combine data from the three RCTs now available on this topic: that by Van Buren *et al*.^82^, the PANDORINA trial^30^, and the PANDRA II trial^81^. The finding that drain omission is associated with reduced POPF rates in LP represents the strongest result of the present analysis, and is supported by high-certainty evidence. A previous meta-analysis by Fatima *et al*.^91^ in 2024 reported reduced rates of POPF and improvements in other postoperative outcomes (including major morbidity, readmission rates, surgical site infections, and LOS) when drains were omitted. Future studies should focus on defining collections after drainless LP as the absence of a drain makes it impossible to diagnose POPF.

The perioperative use of somatostatin analogues to prevent POPF is another important finding of the present study. A 2023 meta-analysis of RCTs by Rompen *et al*.^92^ reported a protective role of perioperative somatostatin analogue administration. However, in sensitivity analyses, performed separately for each analogue in the present study, the benefit was no longer observed when only studies of octreotide were included, indicating that the pooled effect is largely driven by pasireotide. Therefore, the choice between somatostatin analogues remains unclear. In 2024, Gaujoux *et al*.^93^ published an RCT comparing somatostatin and octreotide in the prevention of POPF after pancreatectomy, and reported no significant difference between the groups. In the present meta-analysis, the 2014 single-centre RCT by Allen *et al*.^37^ carried the greatest weight in the pooled result as it reported a clear reduction in POPF with pasireotide compared with placebo following partial pancreatectomy (OR 0.42, 95% c.i. 0.20 to 0.87). The results of this study, however, have not been replicated in a multicentre setting, and concerns remain regarding the cost, efficacy, and availability of pasireotide, which have hampered the implementation of this strategy^94–96^. Additionally, standardization of the timing of somatostatin analogue administration is lacking (for example perioperative *versus* postoperative), further hampering the implementation of this strategy. Taken together, these findings indicate that pasireotide may reduce POPF, whereas evidence remains weak for older analogues such as octreotide. For these reasons, the use of somatostatin analogues cannot yet be recommended as a uniform class effect, and further high-quality, analogue-specific RCTs are warranted.

The present meta-analysis confirmed the effect of perioperative corticosteroid administration in preventing POPF, although the certainty of evidence was low according to GRADE in both the overall analysis and the hydrocortisone-only subgroup. Conversely, when focusing on only on PD, no statistical significance was found, as reported previously by Liu *et al*.^97^. Nonetheless, because the direction of the effect remained favourable, it is reasonable to expect that additional RCTs on the topic will confirm the protective role of this intervention on POPF. Importantly, hydrocortisone and dexamethasone differ substantially in potency and glucocorticoid/mineralocorticoid profile, and pooling results from their use may obscure clinically meaningful differences. As observed with somatostatin analogues, the apparent benefit seems to be driven primarily by a specific agent (hydrocortisone), whereas the evidence supporting others (such as dexamethasone) is weaker. This pattern suggests a drug-specific rather than a true class effect. The lack of statistical significance in PD-specific analysis further indicates that current evidence is not yet robust enough to support routine use in this setting, and additional procedure-specific RCTs are warranted.

Focusing on pancreatic parenchymal characteristics is also pivotal. Based on pancreatic texture and MPD diameter, the ISGPS^98^ recently proposed a risk stratification system (class A, B, C, D) for patients undergoing pancreatic PD. This scenario introduced the concept of a ‘mitigation strategy’ for high-risk patients. For this reason, it seems surprising that less than 10.0% of the RCTs focused specifically on patients at high risk of POPF. Focusing on this category would seem reasonable given that it would require smaller sample sizes to obtain sufficient power.

Future RCTs are also needed among patients with ongoing POPF because the present meta-analysis showed that only four RCTs focused on this topic, and none identified a potential effective strategy. Currently, the management of POPF is not standardized, with available strategies ranging from enteral nutrition or total parenteral nutrition (TPN) and antibiotic therapy to percutaneous catheter drainage, as well as endoscopic, radiological, or surgical reinterventions, aimed at preventing or treating PPH and other septic complications. In 2011, Klek *et al*.^99^ published a RCT that identified enteral nutrition as an effective strategy to treat ongoing POPF compared with TPN. However, this trial was not included in the present meta-analysis because it did not focus only on pancreatic resections. Owing to the substantial lack of evidence on this topic, standardization of POPF management remains a distant goal. Future high-quality studies on this topic must be performed to enable development of evidence-based guidelines for POPF management.

Only one intervention achieved a high certainty of evidence according to the GRADE framework, whereas most interventions showing a statistically significant reduction in POPF had only low-to-moderate certainty. This primarily reflects structural limitations of the underlying RCTs rather than shortcomings of the present analysis. Risk of bias, clinical heterogeneity in the interventions evaluated, and variability in surgical techniques across trials conducted over two decades of evolving pancreatic surgery were the main reasons for downgrading. Moreover, most RCTs enrolled heterogeneous patient populations without stratification by fistula risk, limiting the applicability of pooled estimates to specific clinical scenarios.

Sensitivity analyses further clarified the certainty of evidence. Low certainty was observed in three analyses. For perioperative corticosteroids, downgrading was driven by indirectness and imprecision owing to heterogeneous regimens, different surgical procedures, and limited sample size. Hydrocortisone-only analysis was also rated as having low certainty because of the small number of studies and mixed surgical settings. For PG *versus* PJ, certainty was downgraded because of risk of bias and inconsistency after exclusion of the trial that used a different reconstruction technique. Overall, the available evidence remains immature, highlighting the need for adequately powered, methodologically rigorous trials in well defined patient populations with standardized interventions.

The interventions yielding negative pooled results should not be interpreted as being definitively ineffective. Several strategies, such as early drain removal (OR 0.39), showed effect sizes suggestive of potential benefit, and the supplementary sensitivity analyses indicate that study heterogeneity may have obscured true advantages. As such, the appropriate interpretation is that current evidence remains inconclusive rather than negative. The question of how confident one can be that these interventions do not prevent POPF therefore remains open, and it is likely that adequate RCTs, particularly those designed with procedure- and patient-specific criteria, could change the direction or magnitude of future pooled estimates. Surgeons should be encouraged to design RCTs building on the strategies identified in the present review. Moreover, the absence of trials evaluating combinations of interventions, especially those with distinct mechanisms of action, represents an important gap. Future studies exploring multimodal or combined approaches may provide a more effective strategy for reducing POPF than isolated interventions.

The results of the present study should be assessed considering several limitations. First, interventions were included only if identified as effective by at least one RCT. This approach carries the risk of a type II error, missing potentially effective interventions, especially considering the low power of the available RCTs. Although complementary meta-analyses including all interventions with three or more RCTs would offer a more neutral and comprehensive overview, conducting these additional analyses was considered beyond the scope of the present work and would substantially increase its size and complexity. Future studies specifically evaluating interventions with entirely negative RCT portfolios would be valuable to address this gap.

Second, the included RCTs span a 20-year period, during which surgical techniques, perioperative management, and definitions of POPF have evolved. This temporal heterogeneity may have influenced both the absolute POPF rates and the observed effectiveness of individual interventions, although no large differences were observed in the temporal analysis of POPF rates.

Third, the main finding regarding somatostatin analogues and perioperative corticosteroids must be interpreted with caution, given the considerable heterogeneity among the included studies in terms of patient populations, as well as the type and timing of analogue and cortisone administration.

Fourth, the search strategy relied on the Evidence Map of Pancreatic Surgery, a manually curated and continuously updated resource that integrates RCTs from MEDLINE (PubMed), Embase, Cochrane, Web of Science, Scopus, and major trial registries. Although this approach ensures comprehensive coverage of all major databases, the absence of a parallel independent search represents a methodological limitation.

This systematic review identified 18 interventions from 24 RCTs as being effective in reducing POPF after partial pancreatectomy. After meta-analyses, three interventions (drain omission in LP, perioperative somatostatin analogues, and perioperative corticosteroids) remained as effective strategies but with different certainty of evidence in GRADE assessment. This scenario highlights that current evidence is immature, and it is likely that new RCTs will change the outcome of future meta-analyses. Finally, because POPF is a multifactorial entity, a potential future effective treatment strategy could involve combining interventions in RCTs, especially in high-risk subgroups such as in patients undergoing PD with a non-dilated MPD.

## Supplementary Material

zrag088_Supplementary_Data

## Data Availability

The data extracted and analysed originate from previously published studies, all publicly available.
