# Supplementary material for: Preventing pancreatic fistula following pancreatectomy: meta-analysis of randomized clinical trials
Source: BJS Open. 2026 Sep 21;10(5):zrag088. doi: 10.1093/bjsopen/zrag088 (PMC13591280; doi:10.1093/bjsopen/zrag088)
Supplement: Supplementary file 1 [file zrag088_supplementary_data.zip › Supplementary-material.docx]

**^Preventing pancreatic fistula following pancreatectomy: systematic review and meta-analyses of randomized controlled trials^**

Roberto M. Montorsi MD^1,2,3*^, Alessio Marchetti MD^1,4*^, Tessa E. Hendriks MD^2,3,5,6^, Freek Daams PhD^3,7^, Olivier R. Busch PhD ^2,3^, Bas Groot Koerkamp PhD ^8^, Matteo, De Pastena PhD^1^, Hjalmar C. van Santvoort PhD^9^, Bert A. Bonsing PhD^5^, Salvatore Paiella PhD^10,11^, Giuseppe Malleo PhD ^10,11^, Marc G. Besselink PhD ^2,3^, Roberto Salvia PhD ^1,10^
*Shared first authors

**Affiliations:**

^1^ Department of General and Pancreatic Surgery, The Pancreas Institute, University of Verona Hospital Trust, Verona, Italy;

^2^ Amsterdam UMC, location University of Amsterdam, Department of Surgery, Amsterdam, the Netherlands;

^3^ Cancer Center Amsterdam, the Netherlands;

^4^ Department of Surgery, New York University Grossman School of Medicine, New York, USA;

^5^ Department of Surgery, Leiden University Medical Center, Leiden, the Netherlands

^6^ Dutch institute for Clinical Auditing, Leiden, the Netherlands

^7^ Amsterdam UMC, location Vrije Universiteit, Department of Surgery, Amsterdam, the Netherlands;

^8^ Department of Surgery, Erasmus MC Cancer Institute, Rotterdam, the Netherlands.

^9^ Department of Surgery, Regional Academic Cancer Center Utrecht, University Medical Center Utrecht/St. Antonius Hospital Nieuwegein, Nieuwegein, Utrecht, the Netherlands;

^10^ Pancreatic Surgery Unit, Department of Engineering for Innovation Medicine (DIMI), University of Verona, Verona, Italy;

^11^ Pancreatic Surgery Unit, Department of Surgery, Dentistry, Paediatrics and Gynecology, University of Verona, Verona, Italy;

**Corresponding author:**

Prof M. G. Besselink, MD, PhD

Amsterdam UMC, University of Amsterdam,

Department of Surgery, Cancer Center Amsterdam

De Boelelaan 1117 (ZH-7F), 1081 HV Amsterdam, the Netherlands.

E-mail: [m.g.besselink@amsterdamUMC.nl](mailto:m.g.besselink@amsterdamUMC.nl); Telephone: +31-20-4444400

Twitter: @MarcBesselink

**Correspondence during review phase:**

Roberto Maria Montorsi, MD; Email: [montorsi.imd@gmail.com](mailto:montorsi.imd@gmail.com)

Twitter: @R_Montorsi

| **Supplementary Figures** |  |
| --- | --- |
| Supplementary Figure 1. Leave-one-out analysis: Stent vs. no stent, duct-to-mucosa vs invagination PJ, PG vs PJ, sealant use after LP | *pag. 4* |
| Supplementary Figure 2. Leave-one-out analysis: Early vs. late drain removal, drain vs no drain placement in LP, perioperative somatostatin analogues, perioperative cortisone | *pag. 5* |
| Supplementary Figure 3. Forest plots high-risk-of-bias RCTs exclusion: Perioperative somatostatin analogues | *pag. 6* |
| Supplementary Figure 4. Forest plots high-risk-of-bias RCTs exclusion: Stent vs. no stent | *pag. 7* |
| Supplementary Figure 5. Forest plots high-risk-of-bias RCTs exclusion: PG vs. PJ | *pag. 8* |
| Supplementary Figure 6. Forest plots high-risk-of-bias RCTs exclusion: Sealant on the pancreatic stump in LP | *pag. 9* |
| Supplementary Figure 7. Forest plots subgroup analysis: Perioperative somatostatin analogues after pancreatoduodenectomy | *pag. 10* |
| Supplementary Figure 8. Forest plots subgroup analysis: Perioperative somatostatin analogues (only octreotide) | *pag. 11* |
| Supplementary Figure 9. Forest plots subgroup analysis: Perioperative cortisone after pancreatoduodenectomy | *pag. 12* |
| Supplementary Figure 10. Forest plots subgroup analysis: Perioperative hydrocortisone | *pag. 13* |
| Supplementary Figure 11. Forest plots subgroup analysis: Sealant on the pancreatic stump in LP (only Tachosil®) | *pag. 14* |
| Supplementary Figure 12. Forest plots subgroup analysis: Stent vs. no stent (only pancreaticojejunostomy) | *pag. 15* |
| Supplementary Figure 13. Forest plots subgroup analysis: Stent vs. no stent (only external stent) | *pag. 16* |
| Supplementary Figure 14. Forest plots subgroup analysis: Stent vs. no stent (only internal stent) | *pag. 17* |
| Supplementary Figure 15. Forest plots subgroup analysis: PG vs. PJ (exclusion of Andrianello et al.) | *pag. 18* |
| **Supplementary Tables** |  |
| Supplementary Table 1. Risk of bias assessments for trials reporting a statistically significant reduction in POPF and for studies included in the meta-analyses | *pag. 19* |
| Supplementary Table 2. Factors associated with POPF after partial pancreatectomy | *pag. 20* |
| Supplementary Table 3. POPF associated variables for the interventions for which meta-analyses were performed | *pag. 22* |
|  |  |

**Supplementary Figure 1. Leave-one-out analysis: Stent vs. no stent, duct-to-mucosa vs invagination PJ, PG vs PJ, sealant use after LP**

*CI: confidence interval; PG: pancreaticogastrostomy; PJ: pancreaticojejunostomy; LP: left pancreatectomy*

**Supplementary Figure 2. Leave-one-out analysis: Early vs. late drain removal, drain vs no drain placement in LP, perioperative somatostatin analogues, perioperative cortisone**

*CI: confidence interval; PD: pancreatoduodenectomy; LP: left pancreatectomy*

**Supplementary Figure 3. Forest plots high-risk-of-bias RCTs exclusion: Perioperative somatostatin analogues**

*OR: odds ratio*

**Supplementary Figure 4. Forest plots high-risk-of-bias RCTs exclusion: Stent vs. no stent**

*OR: odds ratio*

**Supplementary Figure 5. Forest plots high-risk-of-bias RCTs exclusion: PG vs. PJ**

*OR: odds ratio*

**Supplementary Figure 6. Forest plots high-risk-of-bias RCTs exclusion: Sealant on the pancreatic stump in LP**

*OR: odds ratio*

**Supplementary Figure 7. Forest plots subgroup analysis: Perioperative somatostatin analogues after pancreatoduodenectomy**

*OR: odds ratio*

**Supplementary Figure 8. Forest plots subgroup analysis: Perioperative somatostatin analogues (only octreotide)**

*OR: odds ratio*

**Supplementary Figure 9. Forest plots subgroup analysis: Perioperative cortisone after pancreatoduodenectomy**

*OR: odds ratio*

**Supplementary Figure 10. Forest plots subgroup analysis: Perioperative hydrocortisone**

*OR: odds ratio*

**Supplementary Figure 11. Forest plots subgroup analysis: Sealant on the pancreatic stump in LP** **(only Tachosil®)**

*OR: odds ratio*

**Supplementary Figure 12. Forest plots subgroup analysis: Stent vs. no stent (only pancreaticojejunostomy)**

*OR: odds ratio*

**Supplementary Figure 13. Forest plots subgroup analysis: Stent vs. no stent (only external stent)**

*OR: odds ratio*

**Supplementary Figure 14. Forest plots subgroup analysis: Stent vs. no stent (only internal stent)**

*OR: odds ratio*

**Supplementary Figure 15. Forest plots subgroup analysis: PG vs. PJ (exclusion of Andrianello et al.)**

*OR: odds ratio*

**Supplementary Table 1. Risk of bias assessments for trials reporting a statistically significant reduction in POPF and for studies included in the meta-analyses**

| ***Study*** | ***Bias due to randomization*** | ***Bias due to deviations from intended intervention*** | ***Bias due to missing outcome data*** | ***Bias in measurement of outcome*** | ***Bias in selection of the reported result*** | ***Overall*** |
| --- | --- | --- | --- | --- | --- | --- |
| Cai et al.^65^ | Low | Low | Low | Low | Low | **Low** |
| Singh et al.^66^ | Low | Low | Moderate | Low | Moderate | **Moderate** |
| Bin et al.^67^ | Moderate | Moderate | Low | Moderate | Moderate | **High** |
| Qureshi et al.^68^ | Low | Moderate | Low | Moderate | Moderate | **High** |
| Motoi et al.^19^ | Low | Moderate | Low | Moderate | Low | **Moderate** |
| Kuroki et al.^69^ | Low | Moderate | Low | Moderate | Moderate | **High** |
| Pessaux et al.^70^ | Low | Moderate | Low | Moderate | Low | **Moderate** |
| Winter et al.^60^ | Moderate | Moderate | Moderate | Moderate | Low | **High** |
| Senda et al.^20^ | Low | Moderate | Low | Moderate | Low | **Moderate** |
| Singh et al.^47^ | Low | Moderate | Low | Moderate | Low | **Moderate** |
| Bai et al.^21^ | Low | Low | Low | Low | Low | **Low** |
| El Nakeeb et al.^71^ | Low | Moderate | Low | Moderate | Low | **Moderate** |
| Xu et al.^22^ | Low | Moderate | Low | Moderate | Low | **Moderate** |
| Berger et al.^23^ | Low | Moderate | Low | Moderate | Low | **Moderate** |
| Andrianello et al.^73^ | Low | Moderate | Low | Moderate | Low | **Moderate** |
| Eguchi et al.^74^ | Low | Low | Low | Low | Moderate | **Moderate** |
| Keck et al.^52^ | Low | Low | Low | Low | Low | **Low** |
| Grendar et al.^54^ | Low | Moderate | Low | Low | Low | **Moderate** |
| Figueras et al.^27^ | Low | Moderate | Low | Moderate | Low | **Moderate** |
| Topal et al.^28^ | Low | Moderate | Low | Moderate | Low | **Moderate** |
| Wellner et al.^75^ | Low | Moderate | Low | Moderate | High | **High** |
| Fernández-Cruz et al.^72^ | Low | Moderate | Low | Moderate | Moderate | **High** |
| Mungroop et al.^43^ | Low | Low | Low | Low | Low | **Low** |
| Uranues et al.^76^ | Low | Low | Low | Low | Low | **Low** |
| Jang et al.^40^ | Low | Low | Low | Moderate | Low | **Moderate** |
| Park et al.^39^ | Low | Low | Low | Low | Low | **Low** |
| Sa Cunha et al.^77^ | Moderate | Moderate | Low | Moderate | Low | **High** |
| Carter et al.^78^ | Low | Moderate | Low | Moderate | Moderate | **High** |
| Montorsi et al.^57^ | Moderate | Moderate | Low | Moderate | Low | **High** |
| Dai et al.^79^ | Low | Low | Low | Low | Low | **Low** |
| Dai et al.^32^ | Low | Low | Low | Low | Low | **Low** |
| Dembinski et al.^80^ | Low | Moderate | Low | Moderate | Moderate | **High** |
| Bassi et al.^58^ | Moderate | Moderate | Low | Moderate | Low | **High** |
| Kaiser et al.^81^ | Low | Low | Low | Low | Low | **Low** |
| van Bodegraven et al.^30^ | Low | Low | Low | Low | Low | **Low** |
| van Buren et al.^82^ | Low | Moderate | Low | Moderate | Low | **Moderate** |
| Cao et al*.*^36^ | Low | Moderate | Low | Low | Low | **Moderate** |
| You et al.^83^ | Moderate | Moderate | Low | Moderate | Low | **High** |
| El Nakeeb et al.^84^ | Low | Moderate | Low | Moderate | Low | **Moderate** |
| Kurumboor et al.^85^ | Low | Moderate | Low | Moderate | Low | **Moderate** |
| Allen et al.^37^ | Low | Low | Low | Low | Low | **Low** |
| Fernández-Cruz et al.^86^ | Moderate | Moderate | Low | Moderate | Moderate | **High** |
| Chen et al.^87^ | Moderate | Low | Low | Low | Low | **Moderate** |
| Kant et al.^88^ | Low | Low | Low | Low | Low | **Low** |
| Antila et al.^17^ | Moderate | Low | Low | Low | Low | **Moderate** |
| Laaninen et al.^89^ | Moderate | Low | Low | Moderate | Low | **Moderate** |
| Liu et al.^24^ | Low | Low | Low | Low | Low | **Low** |
| Shao et al.^25^ | Low | Low | Low | Low | Moderate | **Moderate** |
| Serradilla et al.^26^ | Low | Low | Low | Moderate | Low | **Moderate** |
| Yokoyama et al. ^29^ | Moderate | Moderate | Low | Moderate | Moderate | **High** |
| Witzigmann et al.^31^ | Low | Moderate | Low | Moderate | Low | **Moderate** |
| D'Angelica et al.^33^ | Low | Low | Low | Low | Low | **Low** |
| Yamamoto et al.^34^ | Low | Moderate | Low | Moderate | Low | **Moderate** |
| Zhang et al.^35^ | Low | Low | Low | Low | Low | **Low** |
| Perinel et al.^38^ | Low | Moderate | Low | Moderate | Low | **Moderate** |
| Park et al.^39^ | Low | Low | Low | Moderate | Low | **Moderate** |
| Jang et al.^40^ | Low | Low | Low | Moderate | Low | **Moderate** |
| Hamilton et al.^41^ | Low | Low | Low | Low | Low | **Low** |

# Supplementary Table 2. Factors associated with POPF after partial pancreatectomy

| ***Study*** | ***Type of surgery*** | ***Risk factor*** | ***Results*** |
| --- | --- | --- | --- |
| Lin et al.,  2023^42^ | PD | Intra-abdominal infection | OR 20.262 [5.481-74.902], p= 0.000 |
| Mungroop et al.,  2021^43^ | LP | Pancreatic neck thickness MPD | OR 1.19 [1.10-1.30], p< 0.001 OR 1.68 [1.22-2.32], p= 0.007 |
| Landoni et al.,  2022^44^ | LP | Blood transfusion | OR 4.8 [1.2-20], p= 0.032 |
| Di Mola et al.,  2020^45^ | PD | DGE | OR 20.04 [1.92-208.83], p= 0.012 |
| Park et al.,  2020^39^ | PD, LP | Thrombin-coated collagen patch MPD <3 mm Pancreas soft | OR 4.744 [1.172-19.210], p= 0.029 OR 7.120 [1.399-36.241], p= 0.018 OR 6.525 [1.668-25.529], p= 0.007 |
| Kwon et al.,  2019^46^ | PD | Age Pancreas soft | OR 1.061 [1.1015-1.1110], p= 0.028 p< 0.001 |
| Singh et al.,  2018^47^ | PD | T ≤ 2 N0 | RR 0.3 [0.1-0.8], p= 0.01 RR 3.4 [1.3-9.0], p= 0.02 |
| Schindl et al.,  2018^48^ | PD | BMI ≥ 30 MPD ≤ 4 Pancreas soft | OR 5.28 [1.20-23.18], p= 0.027 OR 5.50 [1.84-6.44], p= .002 OR 0.86 [3.41-28.54], p< 0.001 |
| Cecka et al.,  2018^49^ | PD, LP | MPD ≤ 3 Pancreas soft | OR 3.38 [1.8–6.34], p= 0.0001 OR 5.92 [3.2–10.97], p= 0.00001 |
| Jang et al.,  2017^40^ | LP | Age < 60 years | OR 7.23 [1.93-27.12], p= 0.003 |
| Chen et al.,  2017^50^ | MP | Robotic approach Soft pancreatic texture | OR 0.29 [0.09–0.91], p< 0.034 OR 38.34 [4.50-326.60], p< 0.001 |
| Jang et al.,  2016^51^ | PD | Soft pancreatic texture High BMI Non pancreatic disease | OR 2.01 [1.02-3.95], p= 0.043 OR 3.31 [1.69-6.49], p< 0.001 OR 2.45 [1.36-4.42], p= 0.003 |
| Bai et al.,  2016^21^ | PD | Male sex High-risk disease MPD< 2 | RR 5.13 [1.90-13.87], p= 0.001 RR 3.68 [1.67-11.82], p< 0.001 RR 10.52 [1.61–68.86], p= 0.003 |
| Keck et al.,  2016^52^ | PD | Soft pancreas | OR 2.094 [1.145-3.827], p= 0.016 |
| Hassenpflug et al.,  2016^53^ | LP | Age Coverage procedure Resection level in relation to portal vein | OR 0.939 [0.899-0.982], p= 0.0055 OR 0.301 [0.115-0.789], p= 0.0146 OR 0.754 [0.573-0.993], p= 0.0448 |
| Zhang et al.,  2016^35^ | PD | MPD Abdominal infections Seroperitoneum Pneumonia Longer LOS | p= 0.037 p= 0.006 p= 0.010 p= 0.001 p= 0.002 |
| Xu et al.,  2015^22^ | PD | BMI Longer operative time Soft and not dilated MPD Duct-to-mucosa | OR 3.5, p< 0.001 OR 2.6, p< 0.041 OR 0.36, p= 0.014 RR 0.405, p= 0.013 |
| Grendar et al.,  2015^54^ | PD | Soft pancreas | OR 5.89 [1.7-20.39], p= 0.005 |
| Fujii et al.,  2015^64^ | LP | Estimated pancreatic remnant volume | OR 4.16 [2.01-9.53], p= 0.026 |
| Figueras et al.,  2013^27^ | PD | Female sex BMI> 25 EBL> 650 Soft pancreas PJ | RR 0.11 [0.02-0.49], p= 0.004 RR 11.21 [2.46-51.16], p= 0.002 RR 4.98 [1.29-19.24], p= 0.02 RR 15.76 [3-82.88], p= 0.001 RR 3.83 [1.1-13.37], p= 0.035 |
| Motoi et al.,  2012^19^ | PD | BMI Non dilated MPD No stent | RR 11.45, p= 0.008 RR 5.33, p= 0.046 RR 10.38, p= 0.004 |
| Martin et al.,  2013^56^ | PD | Pancreatic texture associated with POPF | p= 0.02 |
| Montorsi et al.,  2012^57^ | LP | Male sex Spleen preservation | p= 0.013 p= 0.04 |
| Bassi et al.,  2010^58^ | PD | Timing of drain removal Unintentional weight decrease > 10% within 6 months before surgery | OR 24, p= 0.0003 OR 4, p= 0.02 |
| Berger et al.,  2009^23^ | PD | Duct-to-mucosa Soft pancreas | 2.4 [1.1-5.4], p= 0.03 3.7 [1.5-8.8], p= 0.003 |
| Poon et al.,  2007^59^ | PD | No stent MPD< 3mm | RR 3.64 [1.3-12.9], p= 0.04 RR 2.6 [1.5-9.5], p= 0.024 |
| Winter et al.,  2006^60^ | PD | Soft pancreas MPD ≥ 5 | OR 8.1, p< 0.5 OR 0.2, p< 0.5 |
| Van Hilst et al.,  2019^61^ | PD | IL-6 IL-8 CRP | AUC: POPF 994 [534–3265], no-POPF 334 [173–704], p=0.003 AUC: POPF 446 [215–773], no-POPF 182 [122–265], p= 0.007 AUC: POPF 625 [554–718], no-POPF 380 [203-520], p<0.001 |

*POPF: postoperative pancreatic fistula; PD: pancreatoduodenectomy; LP: left pancreatectomy; MPD: main pancreatic duct; BMI: body mass index; LOS: length of hospital stays; OR: odds ratio: RR: relative risk; EBL: expected blood loss; PJ: pancreaticojejunostomy; IL-6: interleukin-6; IL-8: interleukin-8; CRP: c-reactive protein; AUC: area under the curve;*

# Supplementary Table 3. POPF associated variables for the interventions for which meta-analyses were performed

| ***Stenting vs no stent*** | **POPF** | | **DGE** | | **PPH** | | **LOS** | | **Mortality** | |
| --- | --- | --- | --- | --- | --- | --- | --- | --- | --- | --- |
|  | ***St*** | ***NSt*** | ***St*** | ***NSt*** | ***St*** | ***NSt*** | ***St*** | ***NSt*** | ***St*** | ***NSt*** |
| Cai et al.^65^ | 3 | 2 | 3 | 7 | 2 | 2 | NA | NA | 1 | 0 |
| Singh et al.^66^ | 3 | 4 | 2 | 2 | 1 | 1 | 18 | 13 | 0 | 0 |
| Bin et al.^67^ | 4 | 3 | 4 | 4 | 1 | 2 | 13.2 | 11.7 | 0 | 0 |
| Qureshi et al.^68^ | 0 | 2 | NA | NA | 0 | 0 | **38.5*** | **31.8*** | 0 | 0 |
| Motoi et al.^19^ | **3*** | **7*** | 6 | 5 | NA | NA | 22 | 22 | 1 | 0 |
| Kuroki et al.^69^ | 5 | 6 | NA | NA | NA | NA | NA | NA | NA | NA |
| Pessaux et al.^70^ | 19 | 29 | **6*** | **22*** | 9 | 11 | 22.9 | 25.7 | 3 | 3 |
| Winter et al.^60^ | 31 | 26 | 16 | 15 | NA | NA | 7 | 8 | 4 | 2 |
| ***Duct-to-mucosa vs invagination*** | **POPF** | | **DGE** | | **PPH** | | **LOS** | | **Mortality** | |
|  | ***DM*** | ***I*** | ***DM*** | ***I*** | ***DM*** | ***I*** | ***DM*** | ***I*** | ***DM*** | ***I*** |
| Senda et al.^20^ | 14 | 6 | 13 | 9 | 1 | 0 | **24*** | **19*** | 0 | 0 |
| Singh et al.^47^ | 16 | 13 | 26 | 18 | 5 | 5 | 14.9 | 16 | 4 | 8 |
| Bai et al.^21^ | **2*** | **12*** | 3 | 6 | 2 | 4 | **13*** | **15*** | 0 | 1 |
| El Nakeeb et al.^71^ | 5 | 2 | 8 | 7 | 3 | 2 | 8 | 8 | 3 | 4 |
| Xu et al.^22^ | **13*** | **1*** | 10 | 7 | 2 | 1 | 17.2 | 16.4 | 1 | 0 |
| Berger et al.^23^ | 14 | 5 | NA | NA | NA | NA | 7 | 7 | 2 | 0 |
| ***PG vs PJ*** | **POPF** | | **DGE** | | **PPH** | | **LOS** | | **Mortality** | |
|  | ***PG*** | ***PJ*** | ***PG*** | ***PJ*** | ***PG*** | ***PJ*** | ***PG*** | ***PJ*** | ***PG*** | ***PJ*** |
| Andrianello et al.^73^ | 18 | 14 | 13 | 13 | 9 | 9 | 23 | 45 | 2 | 2 |
| Eguchi et al.^74^ | 7 | 8 | 2 | 4 | 3 | 1 | 45 | 41 | 0 | 0 |
| Keck et al.^52^ | 34 | 33 | 20 | 21 | **27*** | **16*** | 15 | 16 | 16 | 7 |
| Grendar et al.^54^ | 8 | 6 | NA | NA | NA | NA | 17.4 | 14 | 2 | 1 |
| Figueras et al.^27^ | **7*** | **19*** | 9 | 11 | 11 | 7 | 12 | 15.5 | 3 | 3 |
| Topal et al.^28^ | **13*** | **33*** | **25*** | **13*** | 21 | 17 | 19 | 18 | 4 | 8 |
| Wellner et al.^75^ | 6 | 7 | 14 | 9 | 2 | 1 | 15 | 17 | 1 | 1 |
| Fernández-Cruz et al.^72^ | 2 | 10 | **2*** | **8*** | 1 | 1 | 12 | 16 | 0 | 0 |
| ***Sealant on pancreatic stump*** | **POPF** | | **DGE** | | **PPH** | | **LOS** | | **Mortality** | |
|  | ***S*** | ***NS*** | ***S*** | ***NS*** | ***S*** | ***NS*** | ***S*** | ***NS*** | ***S*** | ***NS*** |
| Mungroop et al.^43^ | 25 | 29 | 4 | 7 | 2 | 6 | **7*** | **8*** | 2 | 6 |
| Uranues et al.^76^ | 26 | 36 | NA | NA | NA | NA | 12 | 13 | 1 | 0 |
| Jang et al.^40^ | **5*** | **15*** | NA | NA | NA | NA | 10 | 9.9 | 0 | 0 |
| Park et al.^39^ | 11 | 15 | NA | NA | 1 | 0 | 9.7 | 10 | 0 | 0 |
| Sa Cunha et al.^77^ | 41 | 33 | NA | NA | 5 | 4 | 17.5 | 14.9 | 1 | 2 |
| Carter et al.^78^ | 9 | 9 | NA | NA | NA | NA | 5 | 5 | 0 | 0 |
| Montorsi et al.^57^ | 2 | 0 | NA | NA | 1 | 4 | 10 | 10 | 0 | 0 |
| ***Early vs. late drain removal*** | **POPF** | | **DGE** | | **PPH** | | **LOS** | | **Mortality** | |
|  | ***ER*** | ***LR*** | ***ER*** | ***LR*** | ***ER*** | ***LR*** | ***ER*** | ***LR*** | ***ER*** | ***LR*** |
| Dai et al.^79^ | 6 | 10 | 6 | 4 | 6 | 5 | **15*** | **16*** | 0 | 0 |
| Dai et al.^32^ | 2 | 0 | 2 | 5 | 0 | 2 | 12.7 | 13.6 | 0 | 0 |
| Dembinski et al.^80^ | 3 | 5 | 11 | 20 | 2 | 2 | **17.8*** | **21*** | 1 | 1 |
| Bassi et al.^58^ | **1*** | **14*** | 2 | 3 | 0 | 1 | **8.7*** | **11.3*** | 0 | 0 |
| ***Drain vs no drain in LP*** | **POPF** | | **DGE** | | **PPH** | | **LOS** | | **Mortality** | |
|  | ***ND*** | ***D*** | ***ND*** | ***D*** | ***ND*** | ***D*** | ***ND*** | ***D*** | ***ND*** | ***D*** |
| Kaiser et al.^81^ | 18 | 26 | 3 | 6 | 4 | 3 | 12 | 12.9 | 0 | 1 |
| van Bodegraven et al.^30^ | **16*** | **39*** | 2 | 5 | 5 | 7 | **6*** | **6*** | 0 | 0 |
| van Buren et al.^82^ | 20 | 31 | NA | NA | NA | NA | **5*** | **5*** | 0 | 2 |
| ***Somatostatin analogue*** | **POPF** | | **DGE** | | **PPH** | | **LOS** | | **Mortality** | |
|  | ***So*** | ***NSo*** | ***So*** | ***NSo*** | ***So*** | ***NSo*** | ***So*** | ***NSo*** | ***So*** | ***NSo*** |
| Cao et al*.*^36^ | **13*** | **25*** | NA | NA | 7 | 12 | 24 | 23 | 0 | 1 |
| You et al.^83^ | 1 | 5 | NA | NA | NA | NA | 13 | 13 | 0 | 0 |
| El Nakeeb et al.^84^ | 4 | 4 | 6 | 9 | 1 | 1 | 9 | 8 | 3 | 3 |
| Kurumboor et al.^85^ | 6 | 10 | 3 | 5 | NA | NA | **11.7*** | **13.2*** | 1 | 1 |
| Allen et al.^37^ | **12*** | **25*** | NA | NA | NA | NA | 8 | 9 | 1 | 1 |
| Fernández-Cruz et al.^86^ | 2 | 3 | 4 | 4 | NA | NA | 11.5 | 12.1 | 0 | 0 |
| **Perioperative cortisone** | **POPF** | | **DGE** | | **PPH** | | **LOS** | | **Mortality** | |
|  | ***C*** | ***NC*** | ***C*** | ***NC*** | ***C*** | ***NC*** | ***C*** | ***NC*** | ***C*** | ***NC*** |
| Chen et al.^87^ | 34 | 41 | 13 | 17 | 7 | 12 | 16.3 | 19.1 | 3 | 3 |
| Kant et al.^88^ | 5 | 9 | 3 | 8 | 3 | 5 | 10 | 11 | 0 | 1 |
| Antila et al.^17^ | **1*** | **6*** | 1 | 1 | 0 | 0 | 8 | 7 | 0 | 0 |
| Laaninen et al.^89^ | 3 | 9 | 8 | 15 | 4 | 8 | 9 | 11 | 0 | 0 |

*POPF: postoperative pancreatic fistula; DGE: delayed gastric emptying; PPH: post-pancreatectomy hemorrhage; LOS: length of hospital stays; St: stent; NSt: no stent; NA: not available; DM: duct-to-mucosa; I: invagination; PG: pancreaticogastrostomy; PJ: pancreaticojejunostomy; S: sealant; NS: no sealant; ER: early removal; LR: late removal; ND: no drain; D: drain; So: somatostatin analogue; NSo: no somatostatin analogue; C: cortisone; NC: no cortisone;*

**Statistically significant results.*
